# Supramolecular Cyclodextrin Nanofibers for Active Food Preservation: Current Trends and Future Perspectives

**DOI:** 10.3390/foods15152688

**Published:** 2026-07-30

**Authors:** Rajaram Rajamohan, Iruthayapandi Selestin Raja

**Affiliations:** 1 School of Chemical Engineering, Yeungnam University, Gyeongsan 38541, Republic of Korea; 2 Department of Cogno-Mechatronic Engineering, Pusan National University, Busan 46241, Republic of Korea; rajaselestin@pusan.ac.kr

**Keywords:** cyclodextrin, electrospinning nanofibers, anti-microbial activity, food preservation, future directions

## Abstract

Cyclodextrin (CD)-based supramolecular nanofibers (NFs) have emerged as an advanced class of multifunctional materials for active food packaging by integrating host–guest supramolecular chemistry with electrospun nanofibrous architectures. The unique hydrophobic cavity and hydrophilic exterior of CDs enable the encapsulation of a wide range of bioactive compounds, including essential oils, natural antioxidants, antimicrobials, and volatile active agents, thereby enhancing their solubility, stability, controlled release, and preservation efficacy. This review comprehensively discusses the molecular structure and inclusion complexation mechanisms of CDs, recent advances in polymer-assisted and polymer-free electrospinning strategies, and the design of CD-based supramolecular nanofibers for food preservation. Particular emphasis is placed on the relationship between fiber morphology, supramolecular interactions, and controlled release behavior, which collectively govern antimicrobial, antioxidant, moisture management, and barrier properties. Recent developments involving biodegradable polymers, hybrid nanofibrous systems, and cyclodextrin-based metal–organic frameworks (CD-MOFs) are critically summarized, highlighting their roles in improving encapsulation efficiency, mechanical stability, and multifunctional performance. The review further compares CD-based nanofibers with other advanced encapsulation technologies, including liposomes, solid lipid nanoparticles, nanostructured lipid carriers, nanoemulsions, polymeric nanoparticles, microspheres, and conventional MOFs, providing a comprehensive evaluation of their loading capacity, release kinetics, scalability, cost, and regulatory suitability for food-contact applications. Representative applications in the preservation of fruits, vegetables, meat, seafood, dairy products, and bakery products demonstrate significant improvements in microbial inhibition, oxidation resistance, ethylene and volatile organic compound adsorption, and shelf-life extension through sustained delivery of natural preservatives. Ultimately, the current challenges, including large-scale manufacturing, long-term stability, regulatory approval, and commercialization, are discussed together with future directions, focusing on smart packaging, stimuli-responsive delivery systems, intelligent sensing, biodegradable multifunctional materials, and sustainable industrial implementation.

## 1. Introduction

Food packaging is essential for food safety, shelf-life extension, supply-chain resilience, and consumer information, while today the urgent needs are reducing environmental impact (plastic waste), adopting circular business models, and deploying active/smart packaging to reduce food loss and improve traceability [1]. Food packaging plays a pivotal role in ensuring food safety and quality by providing effective protection and preservation: it functions as a barrier against mechanical damage, moisture/oxygen ingress, light penetration, and microbial contamination so that food remains intact and safe across storage, transport, and retail environments [2]. Beyond passive protection, packaging contributes to shelf-life extension, thereby directly reducing food waste: modern materials incorporating improved barrier films, modified atmosphere packaging (MAP), and active agents (e.g., antimicrobials, scavengers) slow spoilage and extend freshness along cold chains [3]. The logistic and supply chain role of packaging is equally vital: it facilitates safe long-distance transport, helps to prevent damages, losses, and enables traceability through barcodes, RFID systems, and, increasingly, IoT-enabled smart sensors that monitor environmental parameters in transit [4]. Packaging also acts as the conduit for regulatory and consumer communication: it carries mandatory nutritional facts, allergen warnings, and traceability codes required by legislation [5].

### 1.1. The Need for Food Preservation

The urgency for improved food packaging has intensified in 2024–2025, driven by converging environmental, economic, and technological pressures. Growing emphasis on environmental sustainability and circular economy targets has positioned packaging waste reduction as a policy priority, with governments and multinational buyers mandating reductions in single-use plastics, higher recyclability, and reuse targets through EU regulations and national legislation (Financial Times). Parallel to regulatory action, consumer expectations are rapidly evolving: recent surveys highlight that a majority of consumers prefer eco-friendly packaging and are willing to pay premiums for products marketed as sustainable, leaving brands that lack clear sustainability strategies at significant reputational risk (Shorr Packaging). At the same time, food security and resilience concerns following the pandemic, climate-related disruptions, and global supply chain constraints have amplified the need for packaging that extends shelf life and supports reliable cold-chain logistics; smart packaging technologies offering real-time monitoring of temperature, humidity, and spoilage markers are increasingly seen as operational necessities rather than experimental add-ons [6]. Technological maturation further reinforces this shift, with rapid progress in active and intelligent packaging systems ranging from antimicrobial coatings and oxygen scavengers to essential-oil release matrices, biosensors, and printed electronics transitioning from research prototypes to early commercial deployment [7]. Additionally, tightening regulatory frameworks on labeling, traceability, and extended producer responsibility (EPR) are forcing companies to redesign packaging systems to ensure compliance and to implement take-back or recycling schemes.

### 1.2. Cyclodextrins

Cyclodextrin (CD) based supramolecular NFs are a promising technology for food preservation, offering active packaging solutions with enhanced antimicrobial and antioxidant properties [8,9,10]. CDs are a family of cyclic oligosaccharides formed from starch. Their unique truncated cone-like structure features a hydrophobic inner cavity and a hydrophilic outer surface [11,12,13].

**Figure 1:**
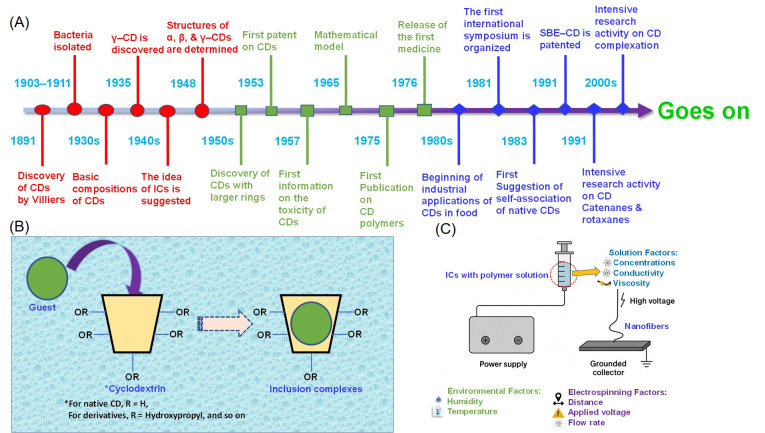
(**A**) A brief development of CDs according to time frame [14] with the permission of the Creative Commons Attribution 4.0 International License (http://creativecommons.org/licenses/by/4.0/). Reprinted with slight modifications, MDPI. (**B**) Schematic representation of guest inclusion in CD cavity for the inclusion complexation. (**C**) Basic setup for the conventional electrospinning process.

This structure allows them to encapsulate hydrophobic “guest” molecules, such as essential oils and other natural preservatives, protecting them from degradation by light, heat, or oxidation. The three most common CDs are α-CD, β-CD, and γ-CD, composed of six, seven, and eight glucose units, respectively. CDs have found extensive applications across diverse fields, including bio-detection, biomedicine, bioimaging, and agricultural production [14]. Over recent decades, advancements in material science have greatly accelerated the development of high-performance CD-based systems (Figure 1A). These efforts have enabled precise regulation of CD structures and have significantly expanded their functional versatility and potential application areas.

### 1.3. Importance of CDs

Among the naturally occurring types α-, β-, and γ-CDs, each differs by the number of glucose units (6, 7, and 8, respectively) and corresponding cavity diameters (approximately 4.7–5.3 Å, 6.0–6.5 Å, and 7.5–8.3 Å, respectively). These differences govern their ability to encapsulate molecules of varying sizes and polarities (Figure 1B). However, native CDs often have solubility limitations, particularly β-CD, leading to the synthesis of modified derivatives such as hydroxypropyl-β-CD (HP-β-CD), methylated β-CD (M-β-CD), and sulfobutylether-β-CD (SBE-β-CD). These derivatives improve aqueous solubility, complexation efficiency, and biocompatibility, expanding their application scope in pharmaceuticals, food, cosmetics, and environmental remediation [15,16,17]. Through inclusion complexation, CDs can enhance the solubility, stability, and bioavailability of guest molecules while protecting them from oxidation, photodegradation, or volatilization. Recent research trends emphasize their role in drug delivery, nanomaterial design, and pollutant removal, aligning with the global demand for sustainable, biodegradable, and eco-friendly materials. Thus, CDs continue to serve as versatile molecular hosts bridging chemistry, biology, and materials science (Table 1) in modern research and industry [18].

### 1.4. Nanofibers

One-dimensional (1D) nanomaterials, particularly NFs with diameters below 1 μm offer a high specific surface area (~40 m^2^/g), very low surface density (<100 mg/cm^2^), and a highly porous structure (>60% porosity, ~10 μm pore size) [39,40]. Early NFs, however, were predominantly fabricated from traditional polymers linked by covalent bonds, limiting their ability to respond rapidly to external stimuli [41]. To address this limitation, stimuli-responsive NFs with tunable and reversible properties have gained significant interest, especially those derived from supramolecular polymers [42]. Unlike conventional polymers, supramolecular polymers are constructed through reversible noncovalent interactions, including hydrogen bonding, host–guest inclusion, ion–dipole interactions, π–π stacking, van der Waals forces, and hydrophobic interactions. These dynamic interactions impart stimuli-responsive behavior, enabling supramolecular systems to adapt to environmental changes and mimic biological recognition and signaling processes [43]. For instance, CD-based supramolecular nanofibers remain structurally stable under ambient conditions but readily dissociate upon heating or exposure to suitable solvents due to the reversible nature of their noncovalent assemblies. This dynamic responsiveness distinguishes CD-based supramolecular NFs from conventional covalently crosslinked polymeric nanofibers, providing unique opportunities for controlled release, self-adaptive functionality, and smart food packaging applications.

Supramolecular-based NFs combine the adaptive responsiveness of supramolecular polymers with the structural advantages of NFs, making them promising functional biomaterials. Nevertheless, achieving controlled and continuous fabrication of such NFs remains challenging. Various methods exist for NF production, including electrospinning [44], self-assembly [45], and wet spinning [46]. Among them, electrospinning is the most widely adopted due to its low cost and versatility [47]. Traditional electrospinning requires high-molecular-weight polymers at relatively high concentrations to ensure sufficient chain entanglement. In contrast, supramolecular-based electrospinning can utilize low-molecular-weight building blocks, as selective supramolecular interactions maintain the necessary solution viscosity for stable fiber formation. As a result, electrospun supramolecular NFs integrate the benefits of both nanofiber technology and dynamic supramolecular chemistry, paving the way for intelligent 1D nanomaterials.

### 1.5. Electrospinning Process

Electrospinning has become one of the most widely used techniques for producing micro- and NFs due to its simplicity, versatility, and low cost. The concept dates back to 1934, when Formhals patented the high-voltage electrostatic spinning method, now recognized as the foundation of modern electrospinning. Since then, extensive studies have explored both the fundamental mechanisms of the electrospinning process and the diverse applications of electrospun ultrafine fibers [47,48,49,50]. Today, electrospinning remains one of the most promising industrial approaches for nanofiber production, capable of processing a wide range of organic polymers, ceramics, and composite materials into fibers with tunable properties. Future advancements are expected to be increasingly interdisciplinary, including integration with supramolecular chemistry.

As illustrated in Figure 1C, electrospinning uses a high-voltage electric field to draw a polymer solution into fine jets that solidify into nanofibers after undergoing rapid, chaotic whipping in air [47]. A typical electrospinning setup consists of a high-voltage power supply, a metal spinneret, and a grounded collector. The spinneret is connected to a syringe pump that delivers the polymer solution, while the collector usually covered with aluminum foil receives the deposited fibers. When high voltage (typically 15–25 kV) is applied, the droplet at the spinneret tip becomes electrically charged, creating a balance between electrostatic repulsion and surface tension. This forms the characteristic Taylor cone. Once the electrostatic forces exceed a critical threshold, a fine jet is ejected. The jet first travels in a stable zone near the Taylor cone, followed by an unstable whipping region as it approaches the collector. During this flight, the solvent evaporates rapidly, resulting in the deposition of solid nanofibers on the collector surface.

The electrospun NFs exhibit unique features that distinguish them from other 1D nanostructures. Firstly, electrospun NFs are extremely long (the length may reach several kilometers in some instances) as electrospinning is a continuous process. Secondly, electrospun NFs possess a high surface area and complex pore structure. In addition, electrospun NFs are products of alignment on the molecular level. The polymer chains experience high shear forces during the electrospinning process and thus become aligned. This makes the electrospun NFs different from solution casting and conventional spinning products. The distinct features of electrospun NFs have attracted many researchers to explore potential applications, such as smart cloth, sensors, electrode materials, enzymes and catalysts, and electronic and optical devices. It is considered that electrospun NFs based on supramolecular interactions will be an interesting subject for further intensive research in the near future.

## 2. Research Methodology

A comprehensive literature review was conducted to collect and analyze information on supramolecular NFs produced via the electrospinning process, incorporating various guest molecules both with and without polymer templates. Relevant scientific articles and books were retrieved from reputable online databases, including Google Scholar, Web of Science, and Scopus, using targeted keyword combinations such as fruit preservation, vegetable preservation, meat preservation, and dairy products.

A systematic selection process was employed to ensure the quality and relevance of the reviewed studies. Initially, all publications referencing food preservation or food packaging were screened for topical relevance. Duplicate entries, non-English publications, and studies outside the research scope were excluded. The remaining articles were critically assessed according to criteria such as the type of food product studied, the nature of the nanofiber materials, the incorporation of ICs, and the use of gels or films. Only studies providing adequate experimental details or comparative performance data were included. Additionally, the reference lists of the selected papers were examined to identify further relevant publications and ensure comprehensive coverage of the topic.

## 3. Anti-Microbial Activities in the Food Packaging Process

The primary goal of food packaging is to preserve the quality, safety, and freshness of food products throughout their shelf life. Traditional packaging acts as a physical barrier against external contaminants (oxygen, moisture, dust, microorganisms) [51]. However, antimicrobial food packaging goes a step further by actively controlling or inhibiting the growth of spoilage and pathogenic microorganisms on the surface or inside the food during storage and distribution. Thus, the antimicrobial activity is directly integrated into the food packaging process to prevent microbial contamination, spoilage (by bacteria, fungi, or yeasts), foodborne illnesses, and sensory and nutritional degradation. A number of anti-microbial agents have been successfully used for the anti-microbial applications (Table 2).

### 3.1. Integration of Antimicrobial Function into Packaging Process

Antimicrobial agents can be incorporated into food packaging through various fabrication or processing techniques, depending on the desired release profile and food compatibility: The following processes are recently used as follows: coating and surface modification (antimicrobial coatings (e.g., chitosan-based films) applied on conventional plastic or paper substrates), blending or extrusion (mixing antimicrobial compounds with polymer melts during extrusion (used in multilayer films), electrospinning (producing nanofiber mats that can encapsulate antimicrobial molecules for controlled release. For example, zein or PVA-based fibers), layer-by-layer assembly (building nanolayers of antimicrobial polymers on packaging surfaces), and encapsulation in nanoparticles or CD complexes (improves stability and controlled diffusion of volatile antimicrobials) [51,56,57,58,59,60].

### 3.2. Benefits of Antimicrobial Packaging

Antimicrobial packaging offers several significant benefits in ensuring food quality, safety, and sustainability [61]. It effectively extends the shelf life of food products by inhibiting or delaying microbial spoilage, thereby preserving freshness over longer storage periods. By actively controlling microbial growth, it also reduces the need for chemical preservatives in food formulations, aligning with consumer demand for more natural and minimally processed foods. Additionally, antimicrobial packaging helps to maintain the sensory quality of food, preserving its color, flavor, and texture by preventing microbial degradation and oxidation. Importantly, this technology enhances food safety by minimizing the risk of contamination from harmful pathogens such as *E. coli*, *L. monocytogenes*, and *S.* spp. Furthermore, when biopolymer-based materials are employed, antimicrobial packaging contributes to sustainable packaging trends, promoting environmental friendliness through biodegradability and reduced plastic waste.

## 4. Anti-Oxidant Activities in the Food Packaging Process

Antioxidant activity in food packaging plays a crucial role in maintaining quality, extending shelf life, and preventing deterioration of food products during storage and transportation [62,63]. Lipid oxidation is a primary cause of spoilage in many foods, particularly those rich in fats leading to off-flavors, color changes, nutrient loss, and reduced safety. The incorporation of antioxidant agents into packaging materials is therefore an effective strategy to minimize oxidative degradation.

The mechanism of action of antioxidant food packaging involves multiple pathways that work together to inhibit or delay oxidative degradation in foods [64,65]. Primarily, incorporated antioxidants act as free radical scavengers, neutralizing reactive oxygen species (ROS) before they interact with lipids, proteins, or other sensitive food components. In addition, certain packaging films function as effective oxygen barriers, reducing oxygen permeability and thereby slowing oxidation-driven spoilage [66]. Packaging films with low oxygen transmission rates (OTR) play a critical role in preserving perishable foods by restricting external oxygen ingress, thereby mitigating oxidative spoilage and enhancing shelf-life stability [67]. Some antioxidants also chelate pro-oxidant metal ions such as Fe^2+^ and Cu^2+^, preventing them from catalyzing oxidative chain reactions [62]. Furthermore, UV-absorbing or light-stabilizing components integrated into the packaging matrix can shield products from photo-oxidation by limiting the penetration of ultraviolet radiation. Hence, these mechanisms contribute to maintaining food quality, extending shelf life, and preserving sensory and nutritional attributes during storage.

Antioxidants can be incorporated into active food packaging systems through various strategies depending on the desired release profile and material functionality. One common approach involves direct blending of antioxidants into biopolymer matrices such as chitosan, gelatin, starch, or polylactic acid (PLA), allowing for uniform distribution throughout the packaging film [68,69]. Alternatively, antioxidants may be deposited as surface coatings to provide rapid interaction with the food surface. Encapsulation techniques, including cyclodextrin complexation and nano-emulsion formation, are often employed to enhance antioxidant stability, protect against volatilization or degradation, and enable controlled release [70]. Electrospinning offers another versatile route, producing nanofiber mats with high surface area that facilitate efficient and responsive antioxidant delivery. Among these strategies, CD-based ICs are particularly advantageous, as they significantly improve the stability of volatile or heat-sensitive antioxidant molecules, enhance their water solubility, and enable tunable release behavior, making them highly suitable for next-generation active packaging applications.

## 5. Shelf Life in the Food Packaging Process After Supramolecular NFs

The incorporation of supramolecular electrospun NFs, particularly those formed from CD ICs, has shown significant potential in extending the shelf life of food products [71]. These NFs act as active packaging components that deliver controlled antioxidant and antimicrobial functions directly at the food–packaging interface. Their high surface area-to-volume ratio enables rapid interaction with oxygen, moisture, and microbial contaminants, thereby slowing spoilage processes. When bioactive compounds such as essential oils, polyphenols, or natural aldehydes are encapsulated within CD cavities and electrospun into NF mats, their volatility is reduced, thermal stability is improved, and release behavior becomes gradual and sustained [72]. This controlled release is critical in maintaining prolonged bioactivity throughout storage rather than causing a rapid initial burst followed by depletion.

## 6. Differences Between Conventional Food Packaging Materials and CD-Based NFs

The key differences between conventional packaging materials and CD-based NFs can be described across several dimensions (Table 3). Traditional packaging systems, although effective in providing mechanical strength and barrier protection, are typically passive and cannot interact actively with food or its surrounding environment [73,74,75,76]. In contrast, CD-based NFs represent an emerging generation of active and intelligent packaging materials that combine the molecular inclusion capability of CDs with the nanoscale structural advantages of electrospun fibrous materials [77]. Conventional packaging materials are generally made from biopolymers, and cellulose materials [78,79]. These materials offer durability, moisture resistance, and good gas barrier properties, but they are inert, non-biodegradable, and contribute to plastic pollution. CD-based NFs, on the other hand, are composed of biodegradable polymers, such as PVA, chitosan, gelatin, or polylactic acid blended with α-, β-, or γ-CDs. Through electrospinning, these blends form highly porous nanofibrous networks with a large surface area-to-volume ratio, allowing the encapsulation of active compounds within the hydrophobic cavity of CDs for functional and responsive packaging performance.

Functionally, conventional materials act as passive barriers, preventing moisture exchange, gas transfer, and physical damage but offering limited biochemical interaction with the food. While some contain additives such as UV blockers or antioxidants, these are merely mixed into the polymer matrix and can migrate or degrade over time. CD-based NFs, however, function as active systems capable of controlled release or absorption of bioactive compounds. Moreover, by incorporating indicator molecules, CD-based NFs can function as intelligent packaging, responding to environmental changes such as pH, temperature, or gas composition.

From a barrier-and-release perspective, conventional packaging offers strong moisture and gas resistance but lacks selective permeability and adaptability. In contrast, CD-based NFs have tunable permeability and can provide sustained release of encapsulated agents triggered by environmental factors like humidity or temperature. Some can even absorb unwanted volatiles such as ethylene or ammonia, thereby slowing spoilage and preserving freshness. In terms of antimicrobial and antioxidant properties, conventional plastics are generally inactive unless coated or doped with external agents, which often lose efficacy over time. CD-based NFs, when loaded with natural bioactives, exhibit potent antimicrobial effects and antioxidant activity, reducing microbial proliferation and lipid oxidation in perishable foods like meat, fish, and dairy.

Environmentally, conventional plastics pose significant sustainability concerns as they are non-biodegradable and may release harmful additives into food. In contrast, CD-based NFs are biodegradable, eco-friendly, and safe, made from renewable sources and incorporating GRAS (Generally Recognized As Safe) components. The encapsulation mechanism within the CD cavity minimizes unwanted migration and ensures controlled, safe interactions with food. Practically, while conventional materials are primarily used for bulk packaging and transport, cyclodextrin-based NFs serve as functional films, coatings, or absorbent pads directly in contact with food surfaces.

In addition, comprehensive comparative information of CD-based nanofibers with other widely investigated encapsulation technologies, including liposomes/nanoliposomes, solid lipid nanoparticles (SLNs), nanostructured lipid carriers (NLCs), nanoemulsions, chitosan/tripolyphosphate nanoparticles, alginate and gelatin microspheres, and conventional metal–organic frameworks (MOFs) is provided in Table S1. It compares those technologies in terms of encapsulation mechanism, loading capacity, release characteristics, structural stability, scalability, cost-effectiveness, regulatory status, and suitability for active food packaging applications.

This comparison emphasizes that CD-based electrospun NFs uniquely integrate molecular host–guest inclusion complexation with a highly porous nanofibrous architecture, enabling simultaneous encapsulation, protection, release, and active packaging functions while maintaining good mechanical properties and high surface area. Unlike lipid-based carriers, which often suffer from limited physical stability and relatively complex preparation, or polymeric micro/nanoparticles that generally require additional incorporation into packaging matrices, CD-based NFs can function directly as active packaging materials with sustained release behavior. Compared with conventional MOFs, CD-based systems also offer improved biocompatibility, lower toxicity concerns, and greater compatibility with food-contact applications.

## 7. Mechanistic Pathways of Food Preservation by CD-Based Supramolecular NFs

**Figure 2:**
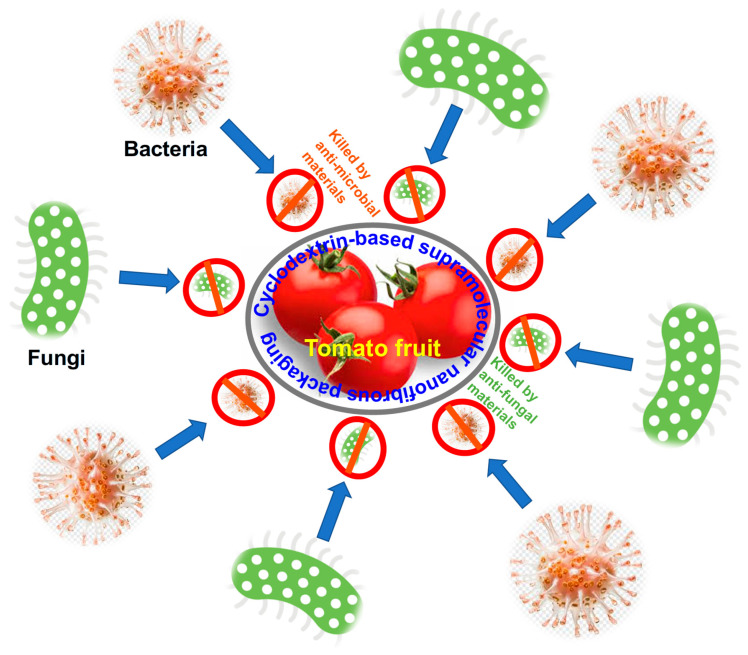
Schematic representation of food samples (ex: tomato fruit) preserved by the packaging with CD-based supramolecular nanofibrous materials.

The protective action of the CD-based supramolecular nanofibrous packaging is primarily governed by the controlled release of encapsulated antimicrobial agents and their subsequent interaction with contaminating microorganisms. The CD cavities stabilize and retain bioactive molecules within host–guest ICs, preventing premature degradation and enabling their gradual diffusion from the nanofibrous matrix when exposed to moisture and temperature fluctuations during storage [80]. As airborne or contact-based bacteria and fungi approach any fruit or meat surface, the porous, high–surface-area NFs facilitate localized release of the active compounds, creating an inhibitory microenvironment around the food samples. The released molecules disrupt microbial cells through multiple pathways, including membrane permeabilization, leakage of intracellular components, inhibition of enzymatic functions, and suppression of spore germination in fungal species. This multilevel antimicrobial action effectively neutralizes the microorganisms before they colonize the fruit surface, as illustrated by the blocked bacterial and fungal penetration events in the schematic. For example, Figure 2 represents the cyclodextrin-based supramolecular nanofibrous packaging for tomato preservation. CD-based ICs enable the sustained release of encapsulated antimicrobial agents, which effectively inhibit bacterial and viral contamination. The electrospun nanofibrous network simultaneously functions as a physical barrier and active delivery platform, thereby preserving fruit quality, enhancing food safety, and extending postharvest shelf life. Hence, the packaging significantly delays microbial spoilage, maintains physicochemical properties, and extends the overall shelf life of the tomatoes.

## 8. Comparison of Existing Reviews and the Present Review

To date, numerous review articles have comprehensively addressed topics related to electrospun nanofibrous materials, CD-based supramolecular assemblies, inclusion complexation, and the biological and environmental applications of supramolecular nanofibrous systems [81,82,83,84,85,86,87,88,89,90,91,92]. Existing reviews have primarily focused on the fundamental chemistry of CDs, electrospinning techniques, host–guest interactions, controlled drug delivery, tissue engineering, environmental remediation, and the general physicochemical characteristics of nanofibrous materials. Several reports have also highlighted the incorporation of CDs into polymeric matrices to improve the stability, solubility, and controlled release of bioactive compounds. Nevertheless, these reviews largely emphasize material synthesis, fabrication methodologies, or specific functional properties, while providing only limited attention to their practical implementation in food preservation and active food packaging.

More importantly, the application of CD-based supramolecular nanofibers in food systems has remained fragmented throughout the literature. Studies involving the preservation of fruits, vegetables, meat, seafood, dairy products, and bakery products are generally discussed independently, without establishing clear relationships between nanofiber design, CD chemistry, encapsulated bioactive compounds, preservation mechanisms, and overall packaging performance. Furthermore, the emerging roles of polymer-free CD nanofibers, crosslinked CD nanofibers, biodegradable biopolymer/CD composites, multifunctional hybrid nanofibers, and CD-based metal–organic framework (CD-MOF) systems have not been critically integrated within a unified framework. Consequently, readers are provided with limited insight into how material composition, supramolecular interactions, electrospinning strategies, and structural design collectively influence antimicrobial activity, antioxidant performance, controlled-release behavior, gas-barrier properties, moisture regulation, intelligent sensing, and shelf-life extension across diverse food matrices.

In addition, previous reviews rarely compare CD-based supramolecular nanofibers with other advanced encapsulation and active packaging technologies, such as liposomes, solid lipid nanoparticles, nanoemulsions, polysaccharide-based nanoparticles, or conventional polymeric packaging systems. As a result, the unique advantages of CD-based NFs, including their high surface-area-to-volume ratio, accessible host–guest inclusion sites, sustained release capability, multifunctionality, and compatibility with biodegradable polymers have not been comprehensively evaluated alongside their current limitations, including production cost, large-scale manufacturing, long-term stability, and regulatory considerations. Likewise, the relationships between NF’s morphology, CD cavity accessibility, pollutant or bioactive interactions, and preservation efficiency remain insufficiently discussed despite their critical importance for designing next-generation food packaging materials.

Therefore, the present review addresses these knowledge gaps by providing a comprehensive and critical synthesis of recent advances in CD-based supramolecular nanofibers for food preservation and active packaging. Unlike previous reviews, this article systematically correlates CD chemistry, supramolecular host–guest interactions, electrospinning technologies, material design strategies, encapsulation mechanisms, controlled-release behavior, and multifunctional preservation performance within a single cohesive framework. Particular emphasis is placed on the preservation of different food categories, including fruits, vegetables, meat and seafood products, by correlating material composition with antimicrobial, antioxidant, moisture-regulating, gas-barrier, and intelligent packaging functions. Furthermore, this review critically discusses the advantages and limitations of various CD-based nanofibrous systems, recent developments in hybrid and multifunctional nanofibers, industrial scalability, regulatory aspects, and future commercialization prospects. By integrating these diverse research areas, this review provides a comprehensive roadmap for the rational design of sustainable, multifunctional, and high-performance supramolecular nanofibrous packaging materials capable of improving food quality, extending shelf life, reducing postharvest losses, and supporting the transition toward environmentally friendly next-generation food packaging technologies.

## 9. Antimicrobial Performance of CD-Based Supramolecular Electrospun NFs

Electrospun NFs incorporating CD ICs have emerged as a transformative platform for antimicrobial delivery, addressing longstanding limitations associated with conventional polymer-based NFs, such as low drug loading, poor stability of hydrophobic or volatile agents, and reliance on toxic organic solvents (Table S2). Across a wide range of studies, CD-based supramolecular NFs consistently demonstrate enhanced antimicrobial efficacy through a unified mechanism that combines high guest loading, amorphous drug dispersion, improved solubility, and rapid or tunable release.

### 9.1. Polymer-Free CD NFs: High Loading and Rapid Antimicrobial Action

A defining advancement in this field is the development of polymer-free electrospun NFs, where CDs act simultaneously as host molecules and fiber-forming matrices. Antibiotic-loaded CD NFs exemplify this strategy, achieving exceptionally high encapsulation efficiencies (45–90%) through inclusion complexation rather than physical entrapment. Structural characterization confirms successful host–guest interactions, evidenced by the disappearance of crystalline drug peaks and the appearance of characteristic antibiotic proton signals. Computational modeling further elucidates these interactions, revealing preferential head-side accommodation of antibiotics within the CD cavity and highlighting drug-dependent complexation energies. Gentamicin, in particular, exhibits the strongest binding affinity in aqueous environments, correlating directly with its superior antibacterial performance against *E. coli*. [93].

Functionally, these polymer-free CD NFs dissolve rapidly in aqueous media and artificial saliva, enabling immediate release of active CD–drug ICs. Disk diffusion assays consistently show pronounced antibacterial zones, with HP-β-CD@gentamicin NFs outperforming kanamycin- and ampicillin-loaded counterparts [93]. This rapid dissolution–high loading synergy positions CD-only nanofibrous mats as promising candidates for fast-acting oral antimicrobial delivery and highlights their potential extension toward antiviral filters and protective textiles.

### 9.2. Antifungal and Agrochemical Applications: Solubility-Driven Bioactivity Enhancement

The same supramolecular principles extend effectively to antifungal and agricultural agents, where poor water solubility often limits biological efficacy. Polymer-free HPβCD/difenoconazole and thiram/HPβCD NFs demonstrate dramatic improvements in solubility, thermal stability, and antifungal activity compared to their free counterparts [94]. Phase solubility studies and molecular docking analyses confirm strong host–guest compatibility, while TGA and XRD data reveal enhanced thermal resistance and amorphous dispersion of the active agents within the fibers. Notably, these NFs dissolve rapidly in water, facilitating prompt release and improved bioavailability of fungicides. The use of water as the sole electrospinning solvent further underscores the environmental sustainability of these systems. Hence, these findings establish CD-based NFs as green, efficient platforms for next-generation pesticide delivery with reduced chemical load and enhanced antifungal performance.

### 9.3. Polymer-Assisted Systems: Controlled Release and Enhanced Residence Time

The functionalization of polymer-free protein-based materials imparts responsiveness to external stimuli, including pH, temperature, humidity, and volatile gas release, enabling real-time monitoring of food freshness and quality [95,96]. Their excellent mechanical integrity and gas-barrier properties effectively retard deterioration processes, thereby enhancing food preservation and extending shelf life [95]. Furthermore, recent advances in fabrication strategies have substantially improved their physicochemical characteristics, including mechanical strength, barrier efficiency, functional stability, and production scalability, highlighting their growing potential as sustainable materials for next-generation active and intelligent food packaging applications.

While polymer-free systems excel in rapid release, polymer-assisted CD NFs introduce an additional level of control over mechanical stability, adhesion, and sustained antimicrobial action. This distinction is clearly illustrated in βCD-based NFs developed for otitis externa treatment. Both polymer-free and polycaprolactone (PCL)-based CD NFs successfully encapsulate ciprofloxacin and dexamethasone in an amorphous state. However, polymer-based systems exhibit superior mechanical robustness, prolonged residence time, and controlled release profiles [97].

In addition, a novel antibacterial packaging material was developed by embedding cinnamon essential oil/β-CD (CEO/β-CD) proteoliposomes into PEO NFs [98]. The nanoliposomes, incorporating proteins within their structure, functioned as proteoliposomes. To evaluate their practical applicability, the antibacterial activity of CEO/β-CD proteoliposome NFs against *B. cereus* on beef was assessed. After 4 days, the NFs achieved nearly a 99.999% reduction in *B. cereus* at 25 °C and 37 °C, and approximately 87.0% and 99.9% reduction at 4 °C and 12 °C, respectively. These results were consistent with the in vitro antibacterial performance and confirm that CEO/β-CD proteoliposome NFs exhibit strong antibacterial efficacy against *B. cereus* on beef.

Surface modification with catechol-functionalized adhesive NFs further enhances mucosal adhesion, allowing extended drug retention in the ear canal. These systems demonstrate an initial burst release followed by sustained antimicrobial delivery, translating into improved bacterial inhibition and reduced dosing frequency. Such findings highlight how polymer incorporation can be strategically employed to tailor antimicrobial release kinetics without compromising the benefits of ICs with CDs.

### 9.4. Natural Antimicrobials and EO Compounds: Stability and Bioactivity Preservation

A major strength of CD-based NFs lies in their ability to stabilize volatile and hydrophobic natural antimicrobials, including ethyl cinnamate, cinnamaldehyde, perillaldehyde, vanillin, linalool, baicalein, quercetin, and plant-derived extracts [99,100,101]. Across these systems, inclusion complexation results in order-of-magnitude improvements in water solubility, substantial enhancement in thermal and photostability, and preservation of antimicrobial functionality.

Ethyl cinnamate, cinnamaldehyde, and perillaldehyde NFs consistently exhibit strong antibacterial activity against both *E. coli* and *S. aureus*, directly linked to their amorphous dispersion and rapid dissolution behavior. Linalool/CD NFs [102] further demonstrate ultra-fast dissolution (<2 s) combined with controlled release under varying environmental conditions, while retaining potent antibacterial effects against Gram-negative and Gram-positive bacteria. In these systems, methylated CDs often provide stronger binding affinities and slower release profiles, illustrating how CD chemistry can be tuned to modulate antimicrobial performance.

### 9.5. Multifunctional Antioxidant–Antimicrobial Systems

Beyond direct antibacterial action, many CD NFs systems exhibit dual antioxidant and antimicrobial functionality, which is particularly advantageous for food preservation, wound care, and biomedical applications. Baicalein [103], quercetin [104], lycopene [105], Vitamin E [106], vanillin [107], and dihydromyricetin-loaded CD NFs [108] demonstrate simultaneous enhancement of solubility, antioxidant capacity, and antimicrobial efficacy. In wound dressing applications, quercetin/CD NFs show rapid release, strong antibacterial inhibition, reduced inflammatory markers, and excellent biocompatibility effects further amplified by chitosan incorporation.

Similarly, CD-based bilayer packaging films incorporating antioxidant ICs effectively suppress microbial growth and oxidative degradation in food systems, outperforming conventional plastic packaging. For example, Zehra et al., 2024 [105], evaluated the antioxidant performance of the NFs of catechin/HP-β-CD NFs with PVA using DPPH* radical scavenging. Scavenging activity increased with catechin concentration, rising from 15.9% at 5 μg mL^−1^ to 76.2% at 40 μg mL^−1^. Incorporating catechin into PVA NFs further enhanced activity; PVA fibers containing 5 μg mL^−1^ catechin showed 20.5% scavenging, exceeding catechin alone. Similar improvements were observed for catechin/HP-β-CD ICs and its respective NFs, both outperforming free catechin at all concentrations. At a concentration of 40 μg mL^−1^, the radical scavenging activity reached 78.6% for PVA NFs, 86.3% for HP-β-CD (IC NFs), and 99.1% for the powder form of HP-β-CD ICs, demonstrating a progressive enhancement in antioxidant performance following inclusion complex formation. The markedly higher scavenging efficiency of the HP-β-CD IC powder suggests that the inclusion complex effectively encapsulates the antioxidant molecules within the hydrophobic cavity of HP-β-CD, thereby improving their aqueous solubility, molecular dispersion, and accessibility to free radicals.

Interestingly, a clear difference in antioxidant activity was observed between the powder and NFs of the HP-β-CD ICs. While the powder form of IC exhibited nearly complete radical scavenging (99.1%), the IC NFs showed a comparatively lower activity (86.3%), although still significantly higher than that of the neat PVA fibers. This reduction may be attributed to the encapsulation of the ICs within the electrospun PVA matrix, which can partially restrict the diffusion and immediate accessibility of antioxidant molecules to DPPH radicals. Furthermore, hydrogen-bonding interactions between PVA and HP-β-CD, together with the dense fibrous architecture, may hinder rapid release of the active compounds, leading to a slower radical-scavenging response during the assay. In contrast, the powdered ICs possess a larger proportion of readily exposed active sites and dissolve or disperse more rapidly in the reaction medium, facilitating more efficient interactions with free radicals. However, no other studies have reported such direct comparisons between the powder and nanofiber forms of similar materials.

### 9.6. Structure–Activity Relationships and Design Principles

Across all systems, a clear structure–activity relationship emerges: stronger host–guest interactions, higher amorphous content, and improved aqueous solubility consistently translate into superior antimicrobial performance. Molecular modeling repeatedly confirms that optimal cavity–guest size matching (e.g., HPγCD for larger molecules, MβCD for volatile aromatics) governs loading efficiency, stability, and release kinetics. Polymer-free systems favor rapid antimicrobial action, while polymer-assisted architectures enable sustained delivery and mechanical durability.

## 10. Supramolecular and Electrospun Nanofibrous Systems for Antimicrobial Food (Fruits and Mushrooms) Preservation

The rapid deterioration of fresh fruits and mushrooms due to microbial spoilage, oxidative stress, and physiological degradation has driven intensive research into active food packaging systems capable of delivering sustained antimicrobial protection. In recent years, electrospun and nanostructured supramolecular systems, particularly those incorporating CD as ICs and bioactive compounds, have emerged as highly effective platforms for food preservation. Hence, the studies reviewed here demonstrate that nanofiber architecture, host–guest complexation, and controlled-release mechanisms act synergistically to enhance antimicrobial efficacy and extend shelf life across diverse fruit and mushroom matrices (Table S3).

### 10.1. Role of CD Inclusion in Enhancing Antimicrobial Stability and Release

A recurring challenge in active food packaging is the volatility, instability, and rapid loss of essential oils (EOs) and natural antimicrobials. Multiple studies consistently demonstrate that cyclodextrin encapsulation, particularly HP-β-CD and β-CD, effectively stabilizes these compounds, significantly enhancing their thermal resistance, solubility, and long-term bioavailability. For example, nerolidol/HP-β-CD NFs [109] exhibited markedly enhanced thermal stability and a 3.3-fold increase in antioxidant activity, while maintaining strong antibacterial effects against *E. coli* and *S. aureus*. This stabilization translated directly into substantial inhibition of *B. cinerea* on strawberries, with lesion diameters reduced by more than fivefold.

Similarly, 1,8-cineole encapsulated in HP-β-CD and embedded within PVA/chitosan NFs demonstrated superior physicochemical performance, including enhanced hydrophobicity, mechanical strength, and light-barrier properties [110]. Importantly, the dual encapsulation strategy (molecular inclusion + nanofiber entrapment) enabled a non-Fickian, sustained release of cineole, ensuring prolonged antimicrobial and anti-browning activity and extending strawberry shelf life to six days under ambient conditions. These findings align with those reported for β-CD encapsulated oregano [111], thyme [112], cinnamon [113], magnolia [114], and lemongrass EOs [115], confirming that CD host–guest interactions are central to preserving EO functionality during storage with a variety of fruits like Strawberry, blackberry, mango, cherry tomato, and so on.

In particular, as an example, blackberry fruits are highly prone to postharvest deterioration, including rapid softening, decay, and pigment degradation. Blackberries coated with the OEO@β-CDs/PLA/PCL NFs retained a firm texture and uniform dark coloration throughout the storage period, indicating markedly improved preservation [111]. By contrast, berries treated with PLA/PCL alone and the control (untreated) began to exhibit noticeable reddening of individual drupelets as early as day 2, reflecting early anthocyanin degradation and loss of structural integrity. As storage advanced to day 10, these two groups showed pronounced spoilage characterized by extensive mold growth, severe tissue breakdown, leakage of berry juice, and widespread formation of red, collapsed drupelets. In comparison, the OEO@β-CDs/PLA/PCL treatment effectively suppressed these deterioration phenomena, demonstrating its superior ability to delay decay and maintain the visual and structural quality of blackberry fruit.

Another example is discussed in detail here, as per the results produced by Tamara et al., 2023 [112]. The antimicrobial performance of the three coating formulations was systematically evaluated in triplicate using cherry tomatoes that had been artificially inoculated with fungal spores. The treatments included: (i) an uncoated control, (ii) a control coating composed of chitosan/gelatin (CS/Gel), and (iii) a CS/Gel coating enriched with a β-CD/7% linalool ICs (β-CD/7% LG-IC). To assess the effectiveness of each coating during 20 days of refrigerated storage, the progression of fungal infection was quantified by calculating the total surface area of each tomato (approximated as an ellipsoid) and the corresponding percentage of surface covered by fungal growth. Tomatoes coated with the β-CD/7% LG-IC formulation exhibited a markedly improved appearance throughout storage, demonstrating substantially less fungal contamination compared with both control groups. The incorporation of 7% LG within the CS/Gel matrix imparted a clear and prolonged antifungal effect, with no visible mold growth observed on these samples over the entire storage period. This result confirms, for the first time, that a CS/Gel coating containing β-CD-stabilized linalool can exert a strong fungicidal action on tomato surfaces. The significant suppression of fungal development highlights the potential of this coating system for extending the shelf-life of tomatoes in real-market conditions, particularly in refrigerated display environments where contamination spread is common. Despite the promising antimicrobial efficacy, further work is required to evaluate the influence of the coating on key physicochemical quality attributes of tomatoes (e.g., firmness, color, respiration rate), as well as to assess its performance at larger scales to support commercial adoption.

### 10.2. NFs Architecture as a Driver of Sustained Antimicrobial Performance

Beyond molecular encapsulation, nanofiber morphology and architecture critically influence antimicrobial efficacy. Electrospun systems, owing to their high surface area, tunable porosity, and interconnected fiber networks enable efficient diffusion and sustained release of bioactive compounds. For instance, core–shell PLGA/thymol NFs [116] fabricated via coaxial electrospinning effectively limited thymol volatilization, allowing gradual diffusion into the headspace surrounding strawberries. This mechanism significantly inhibited bacterial, fungal, and yeast growth, resulting in a pronounced extension of shelf life while maintaining biodegradability.

**Figure 3:**
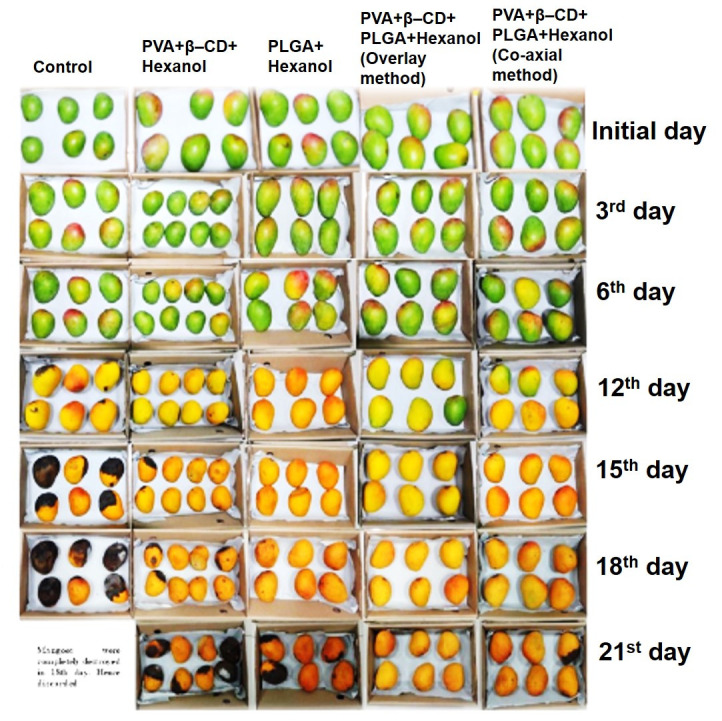
Shelf life studies of mangoes in carton boxes. Reprinted with permission from American Chemical Society [117].

More complex architectures further amplify this effect. Multilayer PVA/β-CD/PLGA NFs matrices loaded with hexanal demonstrated superior release control compared with monolayer or coaxial fibers [117]. Triggered by ambient humidity, these systems released hexanal gradually, modulating phospholipase-D activity and slowing fruit ripening. When applied as “nanostickers” inside mango transport cartons, the multilayer fibers nearly doubled shelf life (23 days vs. 12 days for controls), illustrating how structural design directly governs functional performance. The detailed explanation for the process as follows. A pronounced reduction in physiological weight loss was observed in mango fruits treated with hexanal-loaded nanofiber systems compared with the untreated control (Figure 3). By day 21, fruits exposed to hexanal encapsulated within the PVA+β-CD+PLGA NFs prepared via the overlay method exhibited the lowest weight loss (17.61%), indicating superior moisture retention and delayed senescence. In contrast, control fruits displayed the highest physiological weight loss (26.99%), reflecting accelerated metabolic activity and respiration during ripening. The remaining treatments, PVA+β-CD+hexanal-loaded, PLGA+hexanal-loaded, and PVA+β-CD+PLGA+hexanal-loaded (coaxial method) showed intermediate weight losses of 22.01%, 23.02%, and 20.09%, respectively, on day 21. The substantial weight reduction in the control group is attributable to intensified metabolic changes and rapid water loss driven by elevated respiration rates. Conversely, fruits treated with hexanal-loaded NFs experienced markedly lower weight loss due to hexanal’s inhibitory effect on phospholipase D (PLD), an enzyme implicated in membrane degradation. Suppression of PLD activity helps maintain membrane integrity and promotes cell-wall thickening, thereby reducing moisture loss and enhancing the postharvest stability of mango fruits.

### 10.3. Polymer–CD Synergy and Barrier Enhancement

The integration of biodegradable polymers with CD ICs further enhances antimicrobial effectiveness by improving mechanical integrity, water resistance, and gas-barrier properties. For example, PLA/PCL NFs incorporating OEO@β-CD preserved blackberry quality by maintaining firmness, reducing oxidative stress, and sustaining antioxidant enzyme activity over 10 days of cold storage [111]. Similarly, PCL/PLA NFs containing BHA/HP-β-CD complexes, prepared via ultrasonic mediation, exhibited sustained antioxidant and antimicrobial activity against *E. coli*, *S. aureus*, and *P.* spp., successfully extending mango shelf life [118].

Protein- and polysaccharide-based systems further demonstrate this synergy. Zein/ethyl cellulose hybrid NFs loaded with CEO overcame zein’s inherent water sensitivity through hydrogen bonding interactions, improving hydrophobicity and enabling effective preservation of *Agaricus bisporus* [119]. Likewise, pullulan/γ-CD NFs encapsulating linalool and geraniol achieved exceptional EO retention (up to 77%) and long-term antibacterial activity, underscoring the suitability of edible polymers for food-contact applications [120].

### 10.4. Antimicrobial Efficacy Across Fruits and Mushrooms

Across diverse food systems, strawberries, blackberries, mangoes, kiwifruit, grapes, tomatoes, apples, and mushrooms, the reviewed nanofibrous materials consistently demonstrated broad-spectrum antimicrobial activity, targeting key spoilage organisms such as *B. cinerea*, *P.* spp., *E. coli*, *S. aureus*, yeasts, and molds. Notably, crosslinked PVA/CEO/β-CD NFs significantly delayed mushroom decay while maintaining firmness and color, outperforming conventional PE packaging. In grapes and kiwifruit, alginate- and chitosan-based coatings enriched with CD ICs effectively balanced gas exchange, suppressed microbial growth, and preserved sensory quality without adverse flavor impacts.

As per the results produced by Jiefeng et al., 2019 [115], the hardness of unpackaged mushrooms increased to 210.50 ± 12.71 g after five days, likely due to excessive water loss that slowed internal moisture redistribution. Packaging with the fiber film reduced hardness loss, whereas PE wrap caused a further decline, reaching as low as 93.38 ± 9.55 g by day 5, attributed to restricted moisture exchange that accelerated softening. The colony counts over 1, 3, and 5 days, clearly indicating progressive microbial growth during storage. PE-packaged mushrooms exhibited the highest microbial load, reaching 6.3 ± 0.42 log(CFU/g) on day 5, followed by unpackaged mushrooms at 5.7 ± 0.23 log(CFU/g). Across treatments, total colonies followed the order: CPVA > CPVA-CEO > CPVA-0.5CEO-β-CD > CPVA-1.0CEO-β-CD > CPVA-1.5CEO-β-CD. The incorporation of β-CD enhanced antimicrobial performance, with higher CEO-β-CD concentrations providing stronger inhibition. Mushrooms packaged in PE retained excessive moisture, promoting the growth of spoilage bacteria and resulting in severe blackening by day 5. Samples preserved with CPVA or CPVA-CEO nanofibrous films showed slight discoloration, whereas mushrooms treated with CPVA-CEO-β-CD films remained intact and edible throughout storage.

### 10.5. Emerging Platforms: CD-MOFs and Bio-Nanofibrils

Beyond conventional CD–polymer systems, γ-CD MOFs represent an emerging class of antimicrobial carriers. Limonene-loaded γ-CD-MOFs [121] deposited onto PCL nanofibers achieved high loading capacity (17.6%) and sustained release, resulting in strong antibacterial activity and enhanced preservation of fresh-cut apples. Complementarily, β-lactoglobulin nanofibrils encapsulating resveratrol demonstrated that protein-based supramolecular fibers [122,123] effectively enhance antioxidant stability, inhibit enzymatic browning, and extend the shelf life of sliced apples by approximately 50% compared with the untreated (blank) control, highlighting the versatility of nanofibrillar delivery systems beyond EOs.

## 11. Supramolecular and Nanofibrous Cyclodextrin-Based Systems for Meat Preservation

The preservation of fresh and processed meat products remains a critical challenge due to their high water activity, rich nutrient composition, and susceptibility to microbial spoilage and oxidative degradation. In recent years, CD-based ICs, particularly when integrated into nanofibrous, hydrogel, or polymeric packaging matrices, have emerged as highly effective platforms for extending meat shelf life while addressing limitations associated with volatile natural antimicrobials (Table S4).

### 11.1. Cyclodextrin Encapsulation as a Universal Strategy for Stabilizing Volatile Antimicrobials

A consistent challenge across meat preservation technologies is the high volatility, thermal instability, and strong sensory impact of natural antimicrobials such as cinnamaldehyde [124], citral [124], oregano essential oil (OEO) [125,126], thyme oil [127], carvacrol [128], perilla essential oil [129], and curcumin [130]. Multiple studies demonstrate that β-CD and its derivatives effectively mitigate these issues through host–guest inclusion complexation. The hydrophobic CD cavity encapsulates volatile molecules, protecting them from evaporation and degradation during processing steps such as melt extrusion, electrospinning, or freeze–thaw gelation.

For instance, β-CD encapsulation enabled citral and trans-cinnamaldehyde to withstand high-temperature extrusion into EVOH films without loss of antimicrobial efficacy, a process otherwise incompatible with free EOs [124]. Similarly, OEO/β-CD ICs incorporated into alginate coatings or starch films exhibited enhanced thermal stability, higher encapsulation efficiencies (>70%), and prolonged antimicrobial effectiveness during refrigerated storage of chicken meat [125]. These observations are consistently supported by analytical studies with FT-IR, XRD, SEM, TGA, and DSC measurements, confirming that inclusion complexation alters molecular organization while preserving polymer matrix integrity.

### 11.2. Controlled Release as the Core Mechanism Driving Shelf-Life Extension

Across diverse material platforms, electrospun NFs, edible coatings, nonwoven pads, hydrogels, and composite films, the dominant preservation mechanism is controlled and sustained release of antimicrobials enabled by ICs with CDs. Diffusion studies consistently reveal reduced diffusion coefficients and non-burst release behavior for CD-encapsulated actives compared to free compounds.

In EVOH films, β-CD ICs resulted in a slow migration of citral and cinnamaldehyde, providing continuous antimicrobial protection and extending the chilled beef shelf life by approximately four days [124]. Similarly, sustained cinnamaldehyde release from β-CD-modified PET antibacterial pads [131] prolonged pork freshness by nearly 80%, while maintaining desirable sensory attributes. In hydrogel systems incorporating methyl-β-cyclodextrin/thyme oil complexes [127], diffusion-controlled release followed Fickian kinetics, enabling prolonged antimicrobial activity without rapid volatilization, which resulted in a four-day extension of the shelf life of chilled chicken.

Electrospun nanofiber systems further amplify this effect by combining CD inclusion with high surface area and porous architectures. PLA/perilla essential oil NFs [129], gelatin/pullulan NFs loaded with carvacrol–γ-CD ICs [132], and chitosan/CD films [133] all demonstrated superior antimicrobial longevity due to synergistic regulation of mass transfer at both the molecular and structural levels. To evaluate spoilage, changes in shrimp quality and the corresponding color shift of the colorimetric intelligent film were monitored during storage. The initial TVB-N level was 4.89 mg/100 g and increased steadily to 49.5 mg/100 g by day 7. Similarly, the shrimp’s initial pH of 8.3 rose to 10.5 after 7 days due to tissue decomposition and the formation of nitrogenous compounds. As degradation progressed and volatile nitrogenous substances (e.g., ammonia) accumulated, the film’s color changed from light coral to goldenrod, with its sRGB value increasing to 13.20%. These results demonstrate that the CP/β-CD based colorimetric film is suitable for monitoring shrimp spoilage.

### 11.3. Synergistic Role of Polymer Matrices and CDs

While cyclodextrins govern bioactive stabilization and release, the surrounding polymer matrix critically determines mechanical robustness, barrier performance, and interaction with meat surfaces. Hydrophilic polysaccharides such as alginate, chitosan, carrageenan, and gelatin form semi-permeable films that restrict oxygen and moisture transfer, thereby slowing lipid oxidation and microbial proliferation. In contrast, synthetic biodegradable polymers such as PLA, EVOH, and PVA contribute superior mechanical strength and gas barrier properties, which are essential for industrial packaging applications.

Notably, hybrid systems demonstrate synergistic behavior. In alginate/OEO-β-CD coatings, the alginate matrix provided oxygen and moisture barriers. At the same time, the inclusion complex ensured sustained antimicrobial action, jointly maintaining pH, TVB-N, TBARS, and microbial counts within acceptable limits for up to six days [127]. In PVA-based hydrogels reinforced with polysaccharides and CD inclusion complexes, extensive hydrogen bonding created dense three-dimensional networks with exceptional toughness and elasticity, while simultaneously enabling controlled thyme oil release.

### 11.4. Antimicrobial Spectrum and Sensory Considerations

The reviewed studies consistently report strong inhibition of common meat spoilage and pathogenic microorganisms, including *E. coli*, *S. aureus*, *P. spp.*, lactic acid bacteria, yeasts, and molds. CD-based systems are particularly effective against Gram-positive bacteria, although Gram-negative strains are also significantly suppressed when sustained release is achieved. However, sensory compatibility remains a critical consideration. While CD encapsulation generally reduces aroma intensity, excessive loading or rapid sorption into protein-rich meat matrices can still cause unacceptable flavor changes, as observed in chitosan/CD films loaded with high levels of carvacrol [128,132]. These findings highlight the necessity of optimizing inclusion ratios, film thickness, and release kinetics to balance antimicrobial efficacy with consumer acceptability. Advanced approaches such as photodynamic films [106] (curcumin–β-CD under light activation) offer promising alternatives by achieving strong antimicrobial effects without high volatile concentrations.

### 11.5. Beyond Preservation: Multifunctionality and Intelligent Packaging

Several studies extend the role of CD systems beyond antimicrobial preservation. Intelligent films incorporating β-CD-encapsulated natural pigments exhibited real-time colorimetric responses to spoilage-related volatiles, allowing for the monitoring of freshness alongside antioxidant protection. Similarly, β-CD-enhanced konjac emulsion gels [134] improved structural and rheological properties of low-fat sausages, illustrating how CDs can simultaneously function as stabilizers, encapsulants, and texture modifiers in meat systems.

## 12. Advantages of CD Based Supramolecular NFs

CDs are widely recognized as biocompatible materials, and several native CDs have been accepted for food and pharmaceutical applications because of their favorable toxicological profiles. Although β-CD has been approved for specific food applications in several regulatory jurisdictions, no specific migration limits (SMLs) have currently been established by the U.S. FDA or the European Union for commonly used cyclodextrin derivatives such as HP-β-CD and M-β-CD in food-contact materials. Consequently, the safety evaluation of CD-based active packaging systems should rely on standardized migration testing, toxicological assessment, and compliance with applicable food-contact regulations. In electrospun supramolecular NFs, the incorporation or crosslinking of CDs within polymeric matrices can further minimize CD migration while maintaining controlled release of encapsulated bioactive compounds. Future studies should therefore systematically investigate overall migration, specific migration, and long-term exposure under realistic food storage conditions to facilitate regulatory approval and industrial commercialization.

## 13. Limitations and Challenges of CD Based Supramolecular NFs

Despite the promising potential of CD-based electrospun NFs in food packaging, several limitations and challenges remain. Toxicity concerns persist, as certain CDs, especially at higher concentrations, have been associated with some minimal toxicity. Although this risk is minimal in typical food applications, the use of modified derivatives such as β-CD or γ-CD is generally preferred due to their reduced adverse effects. Scale-up also presents a significant difficulty; while electrospinning offers a versatile and efficient approach for fabricating NF membranes, translating the process into large-scale industrial production requires further technological advancements to ensure uniformity, reproducibility, and cost-effectiveness. Additionally, barrier properties of electrospun NFs can vary depending on some of the parameters, such as polymer composition, fiber morphology, and environmental factors, which in turn affect the efficiency of active packaging. Therefore, optimizing these parameters is essential to achieve consistent performance and meet commercial standards.

## 14. Future Outlook (2026+)

The future of CD-based supramolecular NFs in food preservation is moving toward more sophisticated and multifunctional applications. Advanced formulations are being developed to create multicomponent systems incorporating multiple active compounds, enabling broad-spectrum protection against oxidation, microbial growth, and spoilage. The emergence of smart packaging technologies represents another frontier, where CD-based NFs can be engineered to sense environmental changes/spoilage indicators and release active agents in response to specific prompts, thereby extending shelf life dynamically. Furthermore, bioavailability studies are essential to understand how encapsulated bioactive compounds behave in real food systems, including their release kinetics and effects on sensory. Industrialization efforts will focus on optimizing fabrication techniques such as electrospinning to achieve scalable, cost-effective, and sustainable production, flagging the way for commercial adoption of these innovative materials in the food packaging industry.

## 15. Cost Competitiveness Compared to Conventional Packaging and Commercial Active Packaging Systems

From an economic perspective, the large-scale commercialization of cyclodextrin-based electrospun nanofibers remains more costly than conventional petroleum-based plastic packaging materials, such as PE, PP, and PET, primarily due to the relatively high cost of CDs, bio-based polymers, active compounds, and electrospinning-based fabrication processes. Compared with commercial active packaging systems that typically incorporate low-cost antimicrobial agents or oxygen scavengers into conventional plastic matrices, CD-based nanofibrous systems require additional processing steps and specialized manufacturing equipment, resulting in higher production costs. Nevertheless, these materials offer significant value through their multifunctionality, including controlled release of bioactive compounds, enhanced encapsulation efficiency, improved antioxidant and antimicrobial performance, and reduced reliance on synthetic preservatives, which may offset the initial manufacturing costs in high-value food applications. Furthermore, recent advances in needleless and centrifugal electrospinning, roll-to-roll continuous production, green fabrication processes, and large-scale cyclodextrin production from renewable starch resources are expected to substantially reduce manufacturing costs and improve production efficiency. Future research should therefore focus on techno-economic analysis, life-cycle assessment, process optimization, and scalable manufacturing strategies to establish the commercial viability and cost competitiveness of CD-based nanofibrous packaging relative to existing active packaging technologies.

## 16. Concluding Remarks

Cyclodextrin (CD)-based supramolecular nanofibers have emerged as a versatile platform for next-generation active food packaging by integrating the molecular recognition capability of CDs with the high surface area and porous architecture of electrospun nanofibers. Throughout this review, it is evident that host–guest inclusion complexation serves as the fundamental mechanism for encapsulating, protecting, and controlling the release of bioactive compounds, while the nanofibrous structure enhances mass transfer, loading efficiency, and interaction with food surfaces. The incorporation of natural antimicrobial and antioxidant agents, including essential oils, phenolic compounds, flavonoids, and plant extracts, significantly improves the stability, bioavailability, and sustained release of these bioactives, thereby enhancing antimicrobial efficacy, antioxidant activity, and overall food preservation performance. Recent developments have expanded conventional CD–polymer nanofibers into multifunctional supramolecular systems incorporating biodegradable polymers, protein nanofibers, metal–organic frameworks (CD-MOFs), nanoparticles, and intelligent sensing components. These hybrid materials provide synergistic functionalities such as controlled release, moisture regulation, antimicrobial activity, antioxidant protection, UV shielding, gas adsorption, and freshness monitoring within a single packaging platform. Compared with conventional packaging materials and other encapsulation technologies, CD-based nanofibers offer distinct advantages, including solvent-free encapsulation, high loading efficiency, tunable release kinetics, improved stability of volatile compounds, and environmentally friendly fabrication using biodegradable materials.

Hence, CD-based supramolecular nanofibers represent a highly promising and sustainable platform for active food packaging. Their unique combination of molecular host–guest chemistry, tunable nanofibrous architecture, multifunctional performance, and compatibility with natural bioactive compounds positions them as strong candidates for next-generation food preservation technologies that can simultaneously enhance food safety, extend shelf life, reduce synthetic preservative usage, and minimize environmental impact.

## Figures and Tables

**Table 1:** Key Achievements and challenges of CDs for various applications.

S. No	Application Area	Main Achievements/Focus	Key Challenges/Future Needs	Reference
1	Pharmaceutical Drug Delivery	Enhanced solubility, bioavailability, and stability of poorly soluble drugs; clinical use of HP-β-CD and SBE-β-CD in injectables (e.g., voriconazole)	Toxicological profiling of new CD derivatives; high-purity GMP production; predictable release kinetics	[19,20,21]
2	Nanocarrier & Smart Delivery Systems	Development of CD-based nanoparticles, nanosponges, and CD-MOFs for targeted and stimuli-responsive drug release	Structural optimization for reproducible synthesis; scalable green manufacturing; regulatory approval for advanced formulations	[22,23,24]
3	Cyclodextrin-Metal–Organic Frameworks (CD-MOFs)	High porosity, biocompatible MOFs for controlled drug delivery and gas adsorption	Moisture stability; limited load–release predictability; need for eco-friendly synthesis routes	[25,26,27]
4	Environmental Remediation	β-CD polymers for dye and pesticide adsorption; PFAS removal using CD-based adsorbents	Regenerability of CD adsorbents; cost-effective large-scale deployment; selectivity toward persistent pollutants	[28,29,30]
5	Heavy Metal Recovery/Circular Economy	Phosphorus-functionalized β-CD polymer for selective Sc^3+^ recovery (~98% efficiency)	Adsorption–desorption cycle stability; recyclability without CD degradation	[31,32,33]
6	Food & Nutraceuticals	Stabilization of flavors, vitamins, and antioxidants; masking of bitterness in clean-label foods	Cost-optimization; maintaining “natural” certification; analytical validation of encapsulation efficiency	[34,35]
7	Cosmetic & Personal Care	Sustained release and stabilization of active agents (retinoids, UV filters) in skin formulations	Scale-up and sensory optimization; regulatory compliance for dermal safety	[36,37,38]

**Table 2:** Types of antimicrobial agents used in food packaging.

Category	Examples	Mode of Action	Ref.
Natural agents	Essential oils	Disrupt microbial membranes, inhibit enzyme activity	[52]
Inorganic agents	Silver, zinc oxide, and titanium dioxide nanoparticles	Cell membranes and DNA are damaged by release of metal ions from NPs	[53]
Organically synthesized agents	Sorbates, benzoates, parabens	Inhibit microbial metabolism and reproduction	[54]
Biopolymers	Chitosan, starch, and gelatin	Provide natural antimicrobial and biodegradable matrices	[55]

**Table 3:** Differences between conventional packaging materials and CD-based NFs.

S. No	Features	Conventional Packaging Materials	CD-Based NFs
1	Base Material	Synthetic polymers (PE, PP, PET, PVC)	Biodegradable polymers and β-cyclodextrin
2	Function	Passive barrier	Active & intelligent (controlled release, absorption)
3	Mechanism	Physical protection	Molecular encapsulation and sustained release
4	Antimicrobial/Antioxidant Activity	None or additive-based	Intrinsic via encapsulated natural agents, like drugs
5	Environmental Impact	Non-biodegradable	Biodegradable, eco-friendly
6	Barrier Properties	High but static	Tunable and responsive
7	Food Interaction	Inert	Active interaction (microbial inhibition, oxidation control)
8	Safety	Possible additive migration	GRAS materials, controlled migration
9	Shelf Life Effect	Limited	Prolonged (via microbial and oxidative control
10	Smart Functionality	Absent	Responsive to pH, temperature, or light

## Data Availability

No new data were created or analyzed in this study. Data sharing is not applicable to this article.

## Supplementary Materials

The following supporting information can be downloaded at https://www.mdpi.com/article/10.3390/foods15152688/s1. Table S1: Comparison of cyclodextrin (CD)-based electrospun nanofibers with other advanced encapsulation technologies for active food packaging and controlled delivery applications, Table S2. Comparative summary of CD-based supramolecular electrospun NFs for antimicrobial applications, Table S3. Comparative summary of supramolecular and nanofibrous systems for antimicrobial food (Fruits and Mushrooms) preservation, and Table S4. Comparative summary of supramolecular and nanofibrous systems for antimicrobial food (meats) preservation.

## Abbreviations

CD	Cyclodextrin
β-CD	Beta-Cyclodextrin
HP-β-CD	Hydroxypropyl β-cyclodextrin
M-β-CD	Methylated β-cyclodextrin
γ-CD	γ-cyclodextron
SBE-β-CD	sulfobutylether-β-CD
ICs	Inclusion complexes
NFs	Nanofibers
PVA	Polyvinyl alcohol
PLA	Polylactic acid
PE	Polyethylene
PEO	Polyethylene oxide
PET	Polyethylene terephthalate
PP	Polypropylene
PVC	Polyvinyl chloride.
PCL	Polycaprolactone
PLGA	Poly(lactide-co-glycolide)
PPO	Polyphenol oxidase
TA	Tannic acid
EOs	Essential oils
OEO	Oregano Essential Oil
MOFs	Metal–Organic Frameworks
Ch	Chitosan
CNF	Chitosan nanofibers
Cur	Curcumin
PDI	Photodynamic inactivation
TVC	Total viable count
*E. coli*	*Escherichia coli*
*S. aureus*	*Staphylococcus aureus*
*B. cinerea*	*Botrytis cinerea*
*L. monocytogenes*	*Listeria monocytogenes*
*S.* spp.	*Salmonella* spp.
*B. cereus*	*Bacillus cereus*
*P. citrinum*	*Penicillium citrinum*
*P. aurantiogriseum*	*Penicillium aurantiogriseum*
WVP	Water vapor permeability
CEO	Cinnamon essential oil
T_g_	Glass transition temperature
TVB-N	Total volatile basic nitrogen
EVOH	Ethylene vinyl alcohol

## References

[1] Hu Y., Li T. (2024). Chapter One-Smart food packaging: Recent advancement and trends. Adv. Food Nutr. Res..

[2] Guo J., Rawdkuen S., Al-Gheethi A.A., Ali M., Fei T., Huang Z., Zhang W. (2025). Insights into recent research progress and innovations regarding the application of xanthan gum in the field of food packaging. Food Packag. Shelf Life.

[3] Ahmed M.W., Haque M.A., Mohibbullah M., Khan M.S.I., Mohammed A., Hasan T.M., Raju A. (2022). A review on active packaging for quality and safety of foods: Current trends, applications, prospects and challenges. Food Packag. Shelf Life.

[4] Zhang Z., Xu G., Hu S. (2025). A Comprehensive Review on the Recent Technological Advancements in the Processing, Safety, and Quality Control of Ready-to-Eat Meals. Processes.

[5] Yan M.R., Hsieh S., Ricacho N. (2022). Innovative Food Packaging, Food Quality and Safety, and Consumer Perspectives. Processes.

[6] Yazhiniyan P., Vijayasri K., Nandhini Devi G. (2025). Recent technological advances in food packaging: Sensors, automation, and application. Sust. Food Technol..

[7] Deniz T., Barbera M.K., Hendrik N.J.S. (2024). Food packaging technology considerations for designers: Attending to food, consumer, manufacturer, and environmental issues. Compr. Rev. Food Sci. Food Saf..

[8] Demir S., Celebioglu A., Kayaci-Senirmak F., Ipek O., Uyar T. (2021). Antibacterial Activity of Cyclodextrin-Azo Dye Inclusion Complex Encapsulated Electrospun Polycaprolactone Nanofibers. ChemistrySelect.

[9] Kayaci F., Ozgun C.O.U., Tekinay T., Uyar T. (2013). Antibacterial Electrospun Poly(lactic acid) (PLA) Nanofibrous Webs Incorporating Triclosan/Cyclodextrin Inclusion Complexes. J. Agric. Food Chem..

[10] Aytac Z., Ipek S., Durgun E., Tekinay T., Uyar T. (2017). Antibacterial electrospun zein nanofibrous web encapsulating thymol/cyclodextrin-inclusion complex for food packaging. Food Chem..

[11] Rajamohan R., Mohan S., Sekar A., Choi E.H., Madi F., Le N., Lee Y.R. (2022). Water-soluble inclusion complexes for a novel anti-viral agent with low toxicity; Oseltamivir with the β-cyclodextrins. J. Mol. Liq..

[12] Murugan M., Sivakumar K., Rajamohan R. (2016). A study of host-guest complexation between amodiaquine and native cyclodextrin. Characterization in solid state and its in-vitro anticancer activity. J. Macromol. Chem. A.

[13] Moorthiraman M., Rajaram R., Arumugam A., Madi F. (2022). Non-covalent bonding interaction between primaquine as guest and 2-(hydroxypropyl)-β-cyclodextrin as host. Polycycl. Aromat. Compd..

[14] Zhou J., Jia J., He J., Li J., Cai J. (2022). Cyclodextrin inclusion complexes and their application in food safety analysis: Recent developments and future prospects. Foods.

[15] Mehrdad H., Mohammadi B., Rezaei-Savadkouhi E. (2025). Exploring modified cyclodextrins for enhanced encapsulation and release of ethinyl estradiol: Physicochemical characterization and kinetic modeling. J. Sci. Adv. Mater. Dev..

[16] Chen X., Yang H., Chen S., Yang L., Ren Q., Li Z., Zhang H., Zhang Y. (2021). Solubility and biological activity enhancement of docetaxel via formation of inclusion complexes with three alkylenediamine-modified β-cyclodextrins. RSC Adv..

[17] Angelica S., Marina O., Svetlana B. (2025). Complexation of telmisartan with cyclodextrins as a tool of solubility enhancement: A comparative analysis of influence of the cyclodextrin type and method of solid dispersion preparation. Colloids Surf. A Physicochem. Eng. Asp..

[18] Jitendra W., Niranjan G.K., Sonia G., Swetha R., Abhay P., Rochev Y.A. (2020). Recent advances in host–guest self-assembled cyclodextrin carriers: Implications for responsive drug delivery and biomedical engineering. Adv. Funct. Mater..

[19] Huang J., Wang X., Huang T., Yang Y., Tang J., Zhang J., Yang H., Yang R. (2024). Application of sodium sulfobutylether-β-cyclodextrin based on encapsulation. Carbohydr. Polym..

[20] Rasool F., Naseem A., Javaid H., Afzal Z. (2025). Enhancing the solubility of celecoxib via lyophilized solid dispersions using computational and experimental approaches. Sci. Rep..

[21] Chen W., Liu C., Zhang X., Chen Z., Li W., Bai Y., Wang H., Zhang H., Chen S., Zhang X. (2024). Enhancement of the solubility and oral bioavailability of altrenogest through complexation with hydroxypropyl-β-cyclodextrin. Eur. J. Pharm. Sci..

[22] Yang N., Wang L., Tang Y., Yang P., Xu C., Liu J. (2024). Cyclodextrin-based metal-organic frameworks transforming drug delivery. Eur. J. Med. Chem..

[23] Weng L., Müller K., Möller K., Eickhoff H., Bäumer T. (2025). Cross-linked cyclodextrin-based nanoparticles as drug delivery vehicles: Synthesis strategy and degradation; studies. ACS Omega.

[24] Bose R., Jayawant M., Raut R., Lakkakula J., Roy A., Alghamdi S., Qusty N.F., Sharma R., Verma D., Khandaker M.U. (2023). Cyclodextrin nanoparticles in targeted cancer theranostics. Front. Pharmacol..

[25] Zhao R., Zhu B., Xu Y., Yu S., Wang W., Liu D., Huang J. (2023). Cyclodextrin-based metal-organic framework materials: Classifications, synthesis strategies and applications in variegated delivery systems. Carbohydr. Polym..

[26] Mubarak G.B., Zhang J., Chen L. (2025). Cyclodextrin metal-organic framework design principles and functionalization for biomedical application. Carbohydr. Polym..

[27] Mahya A.M., Károlyi S., Peng F., Nádai M., Kovács J., Rózsa K., Barta P., Marek R., Jankovský O. (2025). Cyclodextrin-Based Metal–Organic Framework as an Application Platform for Bioactive Ruthenium (III) Complexes. Inorg. Chem..

[28] Ana Carolina F.P.F., Sonia J., Luis Felipe O.S., Evandro S.M., Suliman Y.A., Fakhreddine B.A., Tito José C., Samir B., Guilherme L.D. (2025). Novel findings about per- and polyfluoroalkyl substances adsorption on a β-cyclodextrin (β-CD) based adsorbent using the statistical mechanics formalism. Colloids Surf. A Physicochem. Eng. Asp..

[29] Wang R., Lin Z.-W., Klemes M.J., Ateia M., Trang B., Wang J., Cheng C., Helbling D.E., Dichtel W.R. (2022). A Tunable Porous β-Cyclodextrin Polymer Platform to Understand and Improve Anionic PFAS Removal. ACS Cent. Sci..

[30] Dario L., Chiara M., Grégorio C., Nadia M.-C. (2025). Cyclodextrins for the removal of per- and polyfluoroalkyl substances: A review. Environ. Chem. Lett..

[31] Liu K., Song Q., Huang L. (2025). Efficient and rapid recovery of scandium from acidic wastewater using a β-cyclodextrin-based phosphorus-functionalized covalent organic polymer. Nanoscale.

[32] Baatout Z., Jebnouni A., Sakly N., Teka S., Mohamed N., Osman S., Soury R., El Oudi M., Alsaqri S.H., Jaballah N.S. (2025). Phosphonium salt-functionalized β-cyclodextrin film for ultrasensitive and selective electrochemical impedance spectroscopy. Detection of perchlorate in drinking water. Polymers.

[33] Li J., Liu X., Li Q., Xu Z., Zhang Q., Zhang Y., Wang G., Ma B., Xu W. (2025). Separation and recovery of low-concentration rare earth elements from wastewater using a porous magnetic composite of carboxymethyl-β-cyclodextrin and silica. Sep. Purif. Technol..

[34] Gonzalez Pereira A., Carpena M., García Oliveira P., Mejuto J.C., Prieto M.A., Simal Gandara J. (2021). Main Applications of Cyclodextrins in the Food Industry as the Compounds of Choice to Form Host–Guest Complexes. Int. J. Mol. Sci..

[35] Umesh K.V., Aswadh K., Prasanth S., Balu M., Krishnakumar I.M. (2024). Sustained-release microspheres of cyclodextrin–resveratrol complex using fenugreek galactomannan hydrogels: A green approach to phytonutrient delivery. ACS Omega.

[36] Ferreira L., Mascarenhas-Melo F., Rocha S., Maheshwari A., Sharma A., Giram P.S., Pawar K.D., Raza A., Raza F., Veiga F. (2023). Cyclodextrin-based dermatological formulations: Dermopharmaceutical and cosmetic applications. Colloids Surf. B Biointerfaces.

[37] Demirci M.A., Bayrak S.N.K., Demirci N. (2025). Hydroxypropyl-β-Cyclodextrin-enhanced azelaic acid hydrogel for acne treatment: Evaluation of antimicrobial, anti-inflammatory, and skin penetration properties. J. Pharm. Innov..

[38] Pirvu A.S., Varut R.-M., Trasca D.-M., Stoica G.A., Radivojevic K., Carmen S., Arsenie C.C., Popescu C. (2025). Cyclodextrins as active therapeutic agents: Beyond their role as excipients. Pharmaceuticals.

[39] Xia Y., Yang P., Sun Y., Wu Y., Mayers B., Gates B., Yin Y., Kim F., Yan H. (2007). One dimensional nanostructures: Synthesis, characterization, and applications. Adv. Mater..

[40] Greiner A., Wendorff J.H. (2007). Electrospinning: A fascinating method for the preparation of ultrathin fibers. Angew. Chem. Int. Ed..

[41] Huang Z., Zhang Y.Z., Kotaki M., Ramakrishna S. (2003). A review on polymer nanofibers by electrospinning and their applications in nanocomposites. Compos. Sci. Technol..

[42] Webber M.J., Appel E.A., Meijer E.W., Langer R. (2015). Supramolecular biomaterials. Nat. Mater..

[43] Aida T., Meijer E.W., Stupp S.I. (2012). Functional supramolecular polymers. Science.

[44] Wang Y., Luo C., Yang G., Wei X., Liu D., Zhou S. (2016). A luteolin-loaded electrospun fibrous implantable device for potential therapy of gout attacks. Macromol. Biosci..

[45] Zhang S. (2003). Fabrication of novel biomaterials through molecular self-assembly. Nat. Biotechnol..

[46] Meier C., Welland M.E. (2011). Wet-spinning of amyloid protein nanofibers into multifunctional high-performance biofibers. Biomacromolecules.

[47] Li D., Xia Y. (2004). Electrospinning of nanofibers: Reinventing the wheel?. Adv. Mater..

[48] Jung J., Lee C., Yu S., Kim I. (2016). Electrospun nanofibers as a platform for advanced secondary batteries: A comprehensive review. J. Mater. Chem. A.

[49] Zhao W., Li J., Jin K., Liu W., Qiu X., Li C. (2016). Fabrication of functional plga-based electrospun scaffolds and their applications in biomedical engineering. Mater. Sci. Eng. C.

[50] Zhang B., Kang F., Tarascon J., Kim J. (2016). Recent advances in electrospun carbon nanofibers and their application in electrochemical energy storage. Prog. Mater. Sci..

[51] Chen J., Guo R., Zhang Y., Li Q. (2024). Applications of biodegradable materials in food packaging: A review. Alex. Eng. J..

[52] Pal P., Saha T., Dey T., Ihsan I., Sadi M., Zaki S.S., Tan M.Y., Jasmani L., Boon M.D. (2025). Recent Advances in Antimicrobial Food Packaging From Bio-Based Polymers: A Comprehensive Review. J. Appl. Polym. Sci..

[53] Batool A., Abbas G., Ahmed Z., Farooq M., Sajjad H.S., Asif H.M., Bedigama K.K.K.J., Zafar M. (2024). Synthetic vs. natural antimicrobial agents for safer textiles: A comparative review. RSC Adv..

[54] Zhang Y., An J., Shi H., Li B., Liu D., Huang C. (2022). Antimicrobial food packaging integrating polysaccharide-based substrates with green antimicrobial agents: A sustainable path. Food Res. Int..

[55] Cazón P., Morales A.R., Sanches A.S. (2025). Advances in active packaging using natural biopolymers and fruit by-products for enhanced food preservation. Food Res. Int..

[56] Kumar A., Das S., Ali S., Jadhav S.G., Ahmad R., Rahman S.M.E., Ramachandran C., Oh D.-H., Liu S., Wang S. (2025). Mechanisms, applications and challenges of natural antimicrobials in food system. Food Biosci..

[57] Yousefi M., Khorshidian N., Hosseini H. (2020). Potential Application of Essential Oils for Mitigation of Listeria monocytogenes in Meat and Poultry Products. Front. Nutr..

[58] Derya B., Gianmarco I., Gozde S.S., Derya A., Silvia T., Flavia P., Stefano F., Ahmet Y. (2019). Development of flexible antimicrobial zein coatings with essential oils for the inhibition of critical pathogens on the surface of whole fruits: Test of coatings on inoculated melons. Food Packag. Shelf Life.

[59] Rajamohan R., Muhammed A.P., Raorane C.J., Ramasundaram S., Raja I.S., Subramanian S.A., Kim S.C., Oh T.H., Sun S. (2025). Electrospun nanofibers of polyvinylidene fluoride enriched with active antimicrobial tannic acid for the improvement of the shelf life of cherry tomatoes. Materials.

[60] Rajamohan R., Thamaraiselvi K., Raorane C.J., Murugavel K., Govindasamy C., Kim S.-C., Sun S. (2025). Synthesis of the supramolecular structure of vanadium pentoxide nanoparticles with native and modified β-cyclodextrins for antimicrobial performance. Bioengineering.

[61] Khan A.R., Grewal N.S., Sameen D.E., Khan F., Zulqarnain R.M., Zhang H.-J. (2026). Advances in antimicrobial biomaterials for food packaging: A focus on food safety mechanisms and applications. Food Chem..

[62] Karimi I.S., Hosseini B., Hosseini N.R., Moghaddam E., Ebrahimi H., Karimi M., Hosseini D., Hosseini A.S., Karimi A., Gholami H. (2025). Antioxidant packaging films: Application for sustainable food protection. Curr. Res. Food Sci..

[63] Manna S., Gupta M., Das R., Naga Raju M., Pal R. (2025). Sustainable antimicrobial and antioxidant packaging: Environmental implications and solutions. Food Biosci..

[64] Gulcin İ. (2025). Antioxidants: A comprehensive review. Arch. Toxicol..

[65] Samar S., Jafar M.M., Joe M.R., Hossein S.K. (2019). Protection of foods against oxidative deterioration using edible films and coatings: A review. Food Biosci..

[66] Maffioli E., Ruggeri M., Tommasino C., Vigani B., Rossi S., Sandri G. (2026). Beyond Barriers: Active Packaging Strategies for Sustainable Food Protection. Polymers.

[67] Charline R., Jasmine P. (2025). Development of complementary methodologies for oxygen barrier assessment on novel packaging using fluorescent decay and gas chromatography technologies within industrialization environment. Meas. Sens..

[68] Rakesh Kumar G., Manisha, Elsayed A.E.A., Neha N.A., Jyotsana P., Sunil P., Prem P.S., Chin-Kun W., Roberto C.-M. (2026). Vitamin C-infused biopolymer films: A multifunctional approach for active food packaging and preservation. Sustain. Food Technol..

[69] Jessica R.W., Martine W.T., Jiang Y., Zhang X., Andrew D.B., Xie M. (2022). Biodegradable Active Packaging with Controlled Release: Principles, Progress, and Prospects. ACS Food Sci. Technol..

[70] Alieh R.-S., Mahya S., Samira S., Ramin R., Masoumeh A., Ehsan S. (2024). Bioactive compound encapsulation: Characteristics, applications in food systems, and implications for human health. Food Chem. X.

[71] Nabab K., Sudesh Kumar Y., Ankit S. (2025). Development of electrospun pullulan nanofibers encapsulating naringenin/sulfobutylether-β-cyclodextrin inclusion complex for rapid dissolution and antioxidant activity. Int. J. Biol. Macromol..

[72] Peng W., Yan W., Min-Hua Z., Robert J.L., Orcid H.W. (2017). Encapsulation of Bioactive Compound in Electrospun Fibers and Its Potential Application. J. Agric. Food Chem..

[73] Arrieta M.P., Fortunati E., Dominici F., Rayón E., López J., Kenny J.M. (2014). Ternary PLA–PHB–cellulose nanocrystal composites: Thermal, mechanical and disintegration properties. Polym. Degrad. Stab..

[74] Kalpana S., Priyadarshini S.R., Maria Leena M., Moses J.A., Anandharamakrishnan C. (2019). Intelligent packaging: Trends and applications in food systems. Trends Food Sci. Technol..

[75] Atika A., Ahsanulhaq Q., David S. (2023). Nano packaging–Progress and future perspectives for food safety, and sustainability. Food Packag. Shelf Life.

[76] Marsh K., Bugusu B. (2007). Food packaging—Roles, materials, and environmental issues. J. Food Sci..

[77] Adrián M., Silvia N.-O., Francisco G.-C., José Manuel L.-N. (2020). Applications of cyclodextrins in food science. A review. Trends Food Sci. Technol..

[78] Prateek G., Pooja B., Priyanka D., Harish Chandra J., Chauhan P.K., Mikhail S.V., Naveen Chandra J., Anna K., Adarchenko I., Vinod K. (2023). Bio-based food packaging materials: A sustainable and Holistic approach for cleaner environment-A review. Curr. Res. Green Sustain. Chem..

[79] Shabnam S., Hajar S. (2025). Recent trends and advances in biopolymer-based self-healing materials for smart food packaging: A review. Int. J. Biol. Macromol..

[80] Sara P., Amin F., Mehrab P., Majid A. (2024). Cyclodextrin nanocarriers in Coordination Chemistry: Enhancing encapsulation and targeted delivery of 5-Fluorouracil for cancer treatment. Res. Chem..

[81] Fuat T., Tamer U. (2024). Electrospinning of sustainable polymers from biomass for active food packaging. Sustain. Food Technol..

[82] Siddanth S., Manuel J.L. (2025). Native cyclodextrin-based metal–organic frameworks (MOFs): Synthesis, characterization, and potential applications in food industry. Molecules.

[83] Xiao Z., Xu Z., Zhang L., Kang Y., Niu Y., Zhang D. (2025). Application of cyclodextrin-based microcapsules in food flavors and fragrances. Carbohydr. Polym..

[84] Dodero A., Sander G., Heumann A., Vicini S., Castellano M. (2021). Polymer-free cyclodextrin and natural polymer-cyclodextrin electrospun nanofibers: A comprehensive review on current applications and future perspectives. Carbohydr. Polym..

[85] Maria M., Sagitha P., Sujith A. (2025). Biopolymer-based electrospun nanofiber membranes for smart food packaging applications: A review. RSC Adv..

[86] Liu Y., Sameen D.E., Ahmed S., Wang Y., Lu R., Dai J., Li S., Qin W. (2022). Recent advances in cyclodextrin-based films for food packaging. Food Chem..

[87] Harada A., Takashima Y., Yamaguchi H. (2009). Cyclodextrin-based supramolecular polymers. Chem. Soc. Rev..

[88] Friné V.-C., Camilo Z.-L., Iván L.-G., Luis M.-O., Estrella N.-D., José A.G. (2021). Cyclodextrins in polymer-based active food packaging: A fresh look at nontoxic, biodegradable, and sustainable technology trends. Polymers.

[89] Wang Y., Chen J., Sun Y., Wang S., Visser S., Zhang H. (2019). Supramolecular-based nanofibers. Mater. Sci. Eng. C.

[90] Singh S., Mohanty A.K., Dorgan J.R., Copenhaver K., Lozano R., Misra M. (2024). Advances in antimicrobial techniques to reduce postharvest loss of fresh fruit by microbial reduction. npj Sustain. Agric..

[91] Fuat T., Tamer U. (2020). Antioxidant, antibacterial and antifungal electrospun nanofibers for food packaging applications. Food Res. Int..

[92] Zhang C., Li Y., Wang P., Zhang H. (2020). Electrospinning of nanofibers: Potentials and perspectives for active food packaging. Compr. Rev. Food Sci. Food Saf..

[93] Fuat T., Mehmet E.K., Engin D., Gyorgy S. (2021). Fast-dissolving antibacterial nanofibers of cyclodextrin/antibiotic inclusion complexes for oral drug delivery. J. Colloid Interface Sci..

[94] Gao S., Jiang J., Li X., Yang F., Fu Y., Zhao L. (2021). Electrospun Polymer-Free Nanofibers Incorporating Hydroxypropyl-β-cyclodextrin/Difenoconazole via Supramolecular Assembly for Antifungal Activity. J. Agric. Food Chem..

[95] Zubair M., Mortazavi M., Shafique S., Raza Z., Hussain A., Ayyash M., Ullah A. (2025). Exploring protein-based films and coatings for active food packaging applications: A comprehensive review. Int. J. Biol. Macromol..

[96] Lopes A., Medeiros M., Mota I., de Oliveira Sá S., de Paula Rêzende A., Martins G., Carneiro G., Pelissari F.M. (2026). Enhanced Active Packaging Properties Through the Incorporation of Pomegranate Seed Oil Micro- and Nanoparticles. Packag. Technol. Sci..

[97] Hussain Z., Ullah I., Wang Z., Ding P., Ullah S., Zhang Y., Zhang Z., Yang J., Li B., Pei R. (2022). Electrospun nanofibrous membrane functionalized with dual drug-cyclodextrin inclusion complexes for the potential treatment of otitis externa. Colloids Surf. A Physicochem. Eng. Asp..

[98] Lin L., Du Y., Chen H. (2017). Antibacterial poly(ethylene oxide) electrospun nanofibers containing cinnamon essential oil/beta-cyclodextrin proteoliposomes. Carbohydr. Polym..

[99] Subramanian S., Venkatasamy M., Gajanan A.B., Kim M. (2025). Exploring antibacterial ethyl cinnamate/cyclodextrin inclusion complex electrospun nanofibers. J. Mol. Liq..

[100] Gao S., Li X., Yang G., Feng W., Zhang L., Zhao L., Yang F., Fu Y. (2022). Antibacterial perillaldehyde/hydroxypropyl-γ-cyclodextrin inclusion complex electrospun polymer-free nanofiber: Improved water solubility, thermostability, and antioxidant activity. Ind. Crops Prod..

[101] Yildiz M., Engin D., Tamer U. (2019). Electrospun Nanofibers: Fast-Dissolution, Enhanced Water Solubility, High Temperature Stability, and Antibacterial Activity of Cinnamaldehyde. J. Agric. Food Chem..

[102] Zeynep A., Zehra I.Y., Fatma K.-S., Turgay T., Tamer U. (2017). Electrospinning of cyclodextrin/linalool-inclusion complex nanofibers: Fast-dissolving nanofibrous web with prolonged release and antibacterial activity. Food Chem..

[103] Huang X., Wang X., Huang L., Li S., Zhang W. (2021). Antioxidant and antimicrobial polyvinyl alcohol electrospun nanofibers containing baicalein-hydroxypropyl-β-cyclodextrin inclusion complex. Colloids Surf. A Physicochem. Eng. Asp..

[104] Aboelkheir M., Xu R., Kocak M., Chowdhury R., Aboelkheir M., Leung S., Altier C., Bonassar L.J., Shi H., Uyar T. (2024). Antibacterial, anti-inflammatory, and antioxidant cotton-based wound dressing coated with chitosan/cyclodextrin–quercetin inclusion complex nanofibers. ACS Appl. Bio Mater..

[105] Zehra I.Y., Fuat T., Mehmet E.K., Engin D., Tamer U. (2024). Gelatin-based and gelatin-free electrospun fibers of lycopene/cyclodextrin inclusion complexes with potent antioxidant activity. ACS Food Sci. Technol..

[106] Asli C., Tamer U. (2017). Antioxidant Vitamin E/Cyclodextrin inclusion complex electrospun nanofibers: Enhanced water solubility, prolonged shelf life, and photostability of Vitamin E. J. Agric. Food Chem..

[107] Li M., Li X., Zhang R., Zhang K., Ren W., Wang Y., Gao H. (2022). Development and characterization of active bilayer film incorporated with dihydromyricetin encapsulated in hydroxypropyl-β-cyclodextrin for food packaging application. Food Hydrocoll..

[108] Celebioglu A., Kayaci-Senirmak F., Ipek S., Durgun E., Uyar T. (2016). Polymer-free nanofibers from vanillin/cyclodextrin inclusion complexes: High thermal stability, enhanced solubility and antioxidant property. Food Funct..

[109] Lee Y.B., Kim M., Lee Y.J., Shin H., Kang M.H., Kim S. (2025). Cyclodextrins as multifunctional tools for advanced biomaterials in tissue repair and regeneration. Bioact. Mater..

[110] Chen C., Ma T., Li Y., Zhang Y., Yang J. (2023). Electrospun polyvinyl alcohol/chitosan nanofibers incorporated with 1,8-cineole/cyclodextrin inclusion complexes: Characterization, release kinetics and application in strawberry preservation. Food Chem..

[111] Sun C., Fang D., Hu C., Zhang A., Li T., Wang J., Sun Y., Liu L., Wang W., Li W. (2023). Active electrospun nanofiber packaging maintains the preservation quality and antioxidant activity of blackberry. Postharvest Biol. Technol..

[112] Erceg T., Šovljanski O., Stupar A., Ugarković J., Aćimović M., Pezo L., Tomašević A., Tasić M. (2023). A comprehensive approach to chitosan-gelatine edible coating with β-cyclodextrin/lemongrass essential oil inclusion complex. Characterization and food application. Int. J. Biol. Macromol..

[113] Norma E.M., María R.A. (2025). Development and characterization of sustainable active films incorporated with free and microencapsulated thyme essential oil for kiwi-fruit preservation. Front. Sustain. Food Syst..

[114] Liu Y., Wang L., Deng S., Li M., Guo X., Ma F. (2024). Alginate-based edible coating with magnoliae essential oil/β-cyclodextrin inclusion complex for Kyoho grape preservation. LWT-Food Sci. Technol..

[115] Pan J., Ai F., Shao P., Chen H., Gao H. (2019). Development of polyvinyl alcohol/β-cyclodextrin antimicrobial nanofibers for fresh mushroom packaging. Food Chem..

[116] Zhang Y., Zhang Y., Zhu Z., Jiao X., Shang Y., Wen Y. (2019). Encapsulation of Thymol in Biodegradable Nanofiber via Coaxial Electrospinning and Applications in Fruit Preservation. J. Agric. Food Chem..

[117] Preetha S., Kannan M., Subramanian K.S., Govindaraju K. (2024). Encapsulation of Biomolecule (Hexanal) Using Multilayer Electrospun Nanofibers (β-Cyclodextrin/PVA/PLGA) for Controlled Release to Extend the Postharvest Shelf Life of Mango Fruits (Alphonso). ACS Agric. Sci. Technol..

[118] Hu Y., Feng X., Xu H., Yang J., Yang W. (2024). Polycaprolactone/polylactic acid nanofibers incorporated with butyl hydroxyanisole /HP-β-CD assemblies for improving fruit storage quality. Int. J. Biol. Macromol..

[119] Niu B., Zhan L., Shao P., Xiang N., Sun P., Chen H., Gao H. (2020). Electrospinning of zein-ethyl cellulose hybrid nanofibers with improved water resistance for food preservation. Int. J. Biol. Macromol..

[120] Asli C., Emmy H., Mahmoud A., Rimi C., Craig A., Tamer U. (2025). Encapsulation of essential oil-cyclodextrin inclusion complexes in electrospun pullulan nanofibers: Enhanced storage stability and antibacterial property for geraniol and linalool. Food Bioprocess Technol..

[121] Qiu Z., Jiang Q., Zhang Y., Chen M., Li J., Liu Y., Zhang H. (2024). Synthesis of nanosized γ-cyclodextrin metal-organic frameworks as carriers of limonene for fresh-cut fruit preservation based on polycaprolactone nanofibers. Small.

[122] Sivakumar P., Shevchenko V., Laktionov P., Senthilkumar R. (2021). Resveratrol-loaded β-lactoglobulin nanofibrils to prevent enzymatic browning on sliced apple. Appl. Food Biotechnol..

[123] Zhang C., Li L., Liu D., Zhang R., Huang S., Zhang K., Yang B. (2024). Hyaluronic acid-based multifunctional bio-active coating integrated with cinnamaldehyde/hydroxypropyl-β-cyclodextrin inclusion complex for fruit preservation. Int. J. Biol. Macromol..

[124] Chen H., Li L., Ma Y., McDonald T.P., Wang Y. (2019). Development of active packaging film containing bioactive components encapsulated in β-cyclodextrin and its application. Food Hydrocoll..

[125] Yan X., Zhang D., Guo S., Bai P., Xu H., Li Y. (2023). Alginate-based edible coating with oregano essential oil/β-cyclodextrin inclusion complex for chicken breast preservation. Int. J. Biol. Macromol..

[126] Sun Y., Zhang J., Yang C., Cai Y., Yang Y., Zhou C., Wang L., Xia G., Yu X., Yang H. (2022). Preparation and characterization of oregano essential oil-loaded Dioscorea zingiberensis starch film with antioxidant and antibacterial activity and its application in chicken preservation. Int. J. Biol. Macromol..

[127] Chen M., Huang Z., Zhang H., Wu J., Xu X. (2024). Antimicrobial polysaccharide hydrogels embedded with methyl-β-cyclodextrin/thyme oil inclusion complexes for exceptional mechanical performance and chilled chicken breast preservation. Int. J. Biol. Macromol..

[128] Higueras L., López-Carballo G., Hernández-Muñoz P., Catalá R., Gavara R. (2014). Antimicrobial packaging of chicken fillets based on the release of carvacrol from chitosan/cyclodextrin films. Int. J. Food Microbiol..

[129] Wang D., Sun Z., Sun J., Liu F., Du L., Wang D. (2021). Preparation and characterization of polylactic acid nanofiber films loading Perilla essential oil for antibacterial packaging of chilled chicken. Int. J. Biol. Macromol..

[130] Wang J., Liu J., Xu F., Zheng A., Zeng S., Zheng B., Lin S. (2023). Effective preservation of chilled pork using photodynamic antibacterial film based on Curcumin-β-Cyclodextrin complex. Polymers.

[131] Zhang Z., Liu Y., Li Z., Feng L., Dong T., Jin Y., Saldana M.D.A., Sun W. (2020). Sustained-release antibacterial pads based on nonwovens polyethylene terephthalate modified by β-cyclodextrin embedded with cinnamaldehyde for cold fresh pork preservation. Food Packag. Shelf Life.

[132] Ertan K., Celebioglu A., Chowdhury R., Sumnu G., Sahin S., Altier C., Uyar T. (2023). Carvacrol/cyclodextrin inclusion complex loaded gelatin/pullulan nanofibers for active food packaging applications. Food Hydrocoll..

[133] Bakhshpour M., Moghaddam T.N., Tavassoli M., Ayaseh A., Bangar S.P. (2023). Gelatin/chitosan nanofibres containing β-cyclodextrin complex and corn poppy (*Papaver rhoeas* L.) for intelligent packaging. Int. J. Food Sci. Technol..

[134] Kim Y.-J., Shin D.-M., Yune J.-H., Jung H.-S., Kwon H.-C., Lee K.-W., Oh J.-W., Kim B.-G., Han S.-G. (2022). Development of β-cyclodextrin/konjac-based emulsion gel for a pork backfat substitute in emulsion-type sausage. Gels.

