# Supplementary material for: Supramolecular Cyclodextrin Nanofibers for Active Food Preservation: Current Trends and Future Perspectives
Source: Foods. 2026 Jul 30;15(15):2688. doi: 10.3390/foods15152688 (PMC13464602; doi:10.3390/foods15152688)
Supplement: Supplementary file 1 [file foods-15-02688-s001.zip › foods-4397210-supplementary.pdf]

Supramolecular Cyclodextrin Nanofibers for Active Food Preservation: Current Trends and Future Perspectives

Rajaram Rajamohan*[1], Iruthayapandi Selestin Raja[2]

[1]School of Chemical Engineering, Yeungnam University, Gyeongsan 38541, Republic of Korea.

[2]Department of Cogno-Mechatronic Engineering, Pusan National University, Busan 46241, Republic of Korea.

Correspondence: rajmohanau@yu.ac.kr

Table S1. Comparison of cyclodextrin (CD)-based electrospun nanofibers with other advanced encapsulation technologies for active food packaging and controlled delivery applications.

Technology	Encapsulation Mechanism	Loading Capacity	Release Characteristics	Mechanical Stability	Scalability & Cost	Regulatory/ Food Safety	Advantages	Limitations	Typical Applications
Cyclodextrin (CD)-based electrospun nanofibers	Host–guest inclusion complexation within CD cavities combined with electrospun nanofibrous matrix	Moderate–High (depends on CD type and fiber architecture)	Sustained and controlled release through inclusion complexation and diffusion	High after crosslinking; flexible nanofibrous structure	Electrospinning is scalable; moderate production cost	CDs are generally recognized as safe (GRAS); several CD derivatives approved for food and pharmaceutical applications	Simultaneous encapsulation and active packaging, high surface area, excellent VOC adsorption, multifunctionality (antimicrobial, antioxidant, filtration), reusable	Electrospinning optimization required; some biopolymer systems exhibit moisture sensitivity	Active food packaging, wound dressings, drug delivery, air/water purification
Liposomes/ Nanoliposomes	Phospholipid bilayer vesicles	High for hydrophilic and lipophilic compounds	Rapid to moderate release; membrane permeability dependent	Moderate; susceptible to fusion and leakage	Relatively expensive; scale-up can be challenging	Many phospholipids are food-grade and biocompatible	Excellent biocompatibility, suitable for sensitive bioactives	Limited physical stability, oxidation, leakage during storage	Food preservation, nutraceutical delivery, pharmaceuticals
Solid Lipid	Solid lipid	Moderat	Sustained	Good	Industriall	Generally	Protects	Limited	Controlled

Nanoparticles (SLNs)	matrix encapsulation	e	release from solid lipid core		y scalable but relatively costly	regarded as safe using food-grade lipids	sensitive compounds, improved stability over liposomes	drug loading; lipid crystallization may cause expulsion	release, food additives, pharmaceuticals
Nanostructured Lipid Carriers (NLCs)	Mixed solid-liquid lipid matrix	High	Controlled and prolonged release	Good	Scalable; moderate production cost	Uses food-grade lipids	Higher loading capacity than SLNs, reduced drug expulsion	Formulation complexity; stability depends on lipid composition	Functional foods, cosmetics, pharmaceuticals
Nanoemulsions	Dispersion of nanosized oil droplets	Moderate	Rapid release due to large interfacial area	Low–Moderate	Easily scalable; relatively economical	Food-grade emulsifiers widely accepted	Simple preparation, high bioavailability	Poor long-term stability, droplet coalescence and Ostwald ripening	Flavor delivery, essential oils, food preservation
Chitosan/Tripolyphosphate (TPP) nanoparticles	Ionic gelation	Moderate	pH-responsive controlled release	Moderate	Low-cost and simple synthesis	Chitosan is biocompatible and biodegradable	Excellent antimicrobial activity, mucoadhesive properties	Limited loading for hydrophobic compounds; pH sensitivity	Food coatings, drug delivery, antimicrobial packaging
Alginate/Gel	Ionic or	Moderat	Diffusion-	Moderat	Low-cost	Widely	Biodegradab	Lower	Food

atin microspheres	covalent gel formation	e–High	controlled release	e	and highly scalable	accepted biopolymers	le, simple fabrication, good protection of bioactives	mechanical strength; burst release may occur	encapsulation, probiotics, enzyme immobilization
Conventional MOFs (non-CD MOFs)	Adsorption within highly porous crystalline framework	Very High	Controlled release through pore diffusion	High	Often expensive; synthesis may require harsh conditions	Regulatory approval for food applications remains limited	Extremely high surface area and adsorption capacity	Potential metal ion toxicity, complex synthesis, recovery issues	Gas storage, catalysis, pollutant adsorption, drug delivery
CD-MOFs	Combination of CD host–guest chemistry and MOF porosity	High	Controlled release via inclusion and porous diffusion	High	Moderate scalability; more complex than CD polymers	Improved biocompatibility compared with conventional MOFs	High adsorption capacity, molecular selectivity, reusable	More complicated synthesis than CD polymers; relatively high production cost	Drug delivery, pollutant removal, active packaging, sensing

Table S2. Comparative summary of CD-based supramolecular electrospun NFs for antimicrobial applications.

CD Type	Antimicrobial / Bioactive Agent	Fiber System	Loading / Encapsulation Characteristics	Release Behavior	Target Micro-organisms	Key Antimicrobial Outcome	Ref.
HP-β-CD	Gentamicin	Polymer-free CD NF	High loading efficiency (≈45–90%); amorphous dispersion confirmed by XRD; strongest CD–drug interaction (DFT)	Rapid dissolution in water and artificial saliva	*E. coli* (Gram−)	Strongest antibacterial inhibition among the tested Antibiotics	**[93]**
HP-β-CD	Kanamycin	Polymer-free CD NF	High loading via IC formation; reduced crystallinity	Fast dissolution; immediate release	*E. coli*	Effective antibacterial activity, lower than gentamicin	**[93]**
HP-β-CD	Ampicillin	Polymer-free CD NF	Moderate loading; stable IC formation	Rapid release	*E. coli*	Moderate antibacterial performance	**[93]**
HP-β-CD	Difenoconazole	Polymer-free CD NF	IC stoichiometry ~1:0.9; enhanced thermal stability (TGA)	Fast dissolution in aqueous media	Fungal pathogens	Significantly enhanced antifungal activity vs free DZ	**[94]**
HP-β-CD	Thiram	Polymer-free CD NF	High solubility enhancement; amorphous drug state	Rapid water dissolution	Fungal pathogens	Strong antifungal efficacy with reduced chemical usage	**[94]**

β-CD	Ciprofloxacin + Dexamethasone	Polymer-free & PCL-based NF	Amorphous drug distribution; dual-drug encapsulation	Burst + sustained (polymer-based)	*E. coli*, ear canal pathogens	Prolonged antibacterial action; reduced dosing frequency	**[97]**
HP-β-CD	Ethyl cinnamate	Polymer-free CD NF	~10× solubility increase; amorphous EC	Fast dissolution	*E. coli*, *S. aureus*	Enhanced antibacterial efficacy	**[99]**
M-β-CD	Ethyl cinnamate	Polymer-free CD NF	Stronger binding than HP-β-CD; higher thermal stability	Rapid release	*E. coli*, *S. aureus*	Improved antimicrobial potency	**[99]**
HP-γ-CD	Perillaldehyde	Polymer-free CD NF	High encapsulation stability; reduced volatility	Fast dissolution	*E. coli*, *S. aureus*	Strong antibacterial and antioxidant activity	**[100]**
HP-β-CD	Cinnamaldehyde	Polymer-free CD NF	Stable IC (1:1, 2:1); reduced evaporation	Rapid release	*E. coli*	Retained strong antibacterial activity	**[101]**
HP-γ-CD	Cinnamaldehyde	Polymer-free CD NF	Slightly weaker binding vs HP-β-CD	Rapid release	*E. coli*	Effective antibacterial action	**[101]**
HP-β-CD	Baicalein	PVA-based CD NF	Enhanced solubility; improved thermal stability	Sustained release	*E. coli*, *S. aureus*, *Candida albicans*	Strong antioxidant and antimicrobial effects	**[103]**
HP-β-CD / HP-γ-CD	Quercetin	Polymer-free CD NF (cotton-coated)	~20× solubility enhancement; near-complete retention	Fast release	*E. coli*, *S. aureus*	Potent antibacterial and anti-inflammatory effects	**[104]**

HP-β-CD	Vitamin E	Polymer-free CD NF	High loading (~11% w/w); ~83% retention after 3 years	Rapid dissolution	Indirect antimicrobial via antioxidant protection	Long-term stability and preserved antioxidant activity	**[106]**
CD (± gelatin)	Lycopene	CD NF / CD–gelatin NF	Improved UV and thermal stability	Faster dissolution with gelatin	Indirect antimicrobial support	Maintained antioxidant activity	**[100]**
HP-β-CD	Dihydromyricetin	CS/SA bilayer film	Improved mechanical + antimicrobial properties	Sustained release	*Staphylococcus aureus*	Reduced microbial growth in food systems	**[107]**
HP-β-CD / M-β-CD / HP-γ-CD	Vanillin	Polymer-free CD NF	High loading (~12% w/w); volatility preserved	Fast dissolution	Foodborne bacteria	Enhanced antimicrobial and antioxidant effects	**[108]**
HP-β-CD / M-β-CD / HP-γ-CD	Linalool	Polymer-free CD NF	45–89% retention; highest with M-β-CD	Ultra-fast (<2 s) + sustained (M-β-CD)	*E. coli*, *S. aureus*	Strong antibacterial performance	**[102]**

Table S3. Comparative summary of supramolecular and nanofibrous systems for antimicrobial food (Fruits and Mushrooms) preservation.

CD / Host system	Active antimicrobial / bioactive	Nanofiber / coating matrix	Encapsulation & release characteristics	Target microorganisms	Food matrix	Preservation outcomes	Ref.
HP-β-CD	Nerolidol	Electrospun NFs (IC-NF)	Inclusion complex enhanced solubility and thermal stability; sustained diffusion-controlled release	*E. coli*, *S. aureus*, *B. cinerea*	Strawberries	5.56-fold reduction in lesion diameter; strong antioxidant and antibacterial activity; extended shelf life	**[109]**
HP-β-CD	1,8-Cineole	PVA/Chitosan electrospun NFs	Dual encapsulation (IC + nanofiber); non-Fickian prolonged release	General spoilage bacteria, browning-related microbes	Strawberries	Shelf life extended to 6 days at 25 °C; reduced browning and microbial growth	**[110]**
— (polymer barrier)	Thymol	PLGA core–shell NFs (coaxial electrospinning)	PLGA shell suppressed volatilization; slow headspace diffusion	Bacteria, fungi, yeasts	Strawberries	Marked shelf-life extension; strong antimicrobial effect	**[115]**

β-CD	Oregano essential oil	PLA/PCL electrospun NFs	IC-mediated stabilization; controlled release via nanofiber matrix	Oxidative and microbial spoilage flora	Blackberries	Reduced decay (27.8%), weight loss (3.56%); maintained firmness and antioxidants	**[111]**
HP-β-CD	Lemongrass oil	Chitosan/Gelatin edible coating	β-CD improved EO dispersion and long-term release	*P. aurantiogriseum*	Cherry tomatoes	Complete fungal inhibition at 7% IC over 20 days	**[112]**
HP-β-CD	Butyl hydroxyanisole (BHA)	PCL/PLA electrospun NFs	Ultrasonic-assisted IC formation; sustained antioxidant release	*E. coli*, *S. aureus*, *P.* spp.	Mango	Reduced microbial load; extended shelf life to 7 days	**[118]**
β-CD	Hexanal	Multilayer PVA/PLGA electrospun NFs	Overlay electrospinning enabled humidity-triggered controlled release	Ripening-related microbial/enzymatic pathways	Mango	Shelf life extended to 23 days; delayed ripening	**[117]**
β-CD	Thyme essential oil	Chitosan/Gelatin composite films & coatings	Encapsulation slowed EO release; improved barrier properties	Mesophilic bacteria, yeasts, molds	Kiwifruit	Reduced weight loss; ~3 Log CFU g^{-1} microbial reduction	**[113]**

β-CD	Magnolia essential oil	Sodium alginate edible coating	Stable IC with high EE (82.56%); sustained release	*B. cinerea*, *P. citrinum*	Kyoho grapes	Lower decay (16.67%), weight loss (2.21%); preserved firmness	**[114]**
β-CD	Cinnamon essential oil	Crosslinked PVA electrospun NFs	Crosslinking + IC stabilized CEO; long-term antimicrobial release	*E. coli*, *S. aureus*	Mushrooms	Delayed decay; better color and texture vs PE film	**[115]**
Yin— (no CD)	Cinnamon essential oil	Zein/ethyl cellulose electrospun NFs	Hydrogen bonding reduced hydrophilicity; sustained CEO release	General spoilage microbes	Button mushrooms	Reduced weight loss; maintained firmness	**[119]**
γ-CD	Linalool / Geraniol	Pullulan electrospun NFs	γ-CD ensured high EO retention (74–77%); long-term stability	*E. coli*, *S. aureus*	Model food systems	Strong antibacterial activity; high EO preservation	**[120]**
γ-CD-MOF	Limonene	MOF-decorated PCL NFs	High loading (17.6%); MOF-based sustained release	*E. coli*, *S. aureus*	Fresh-cut apples	Inhibited bacterial growth; extended freshness	**[121]**

β-lg nanofibrils	Resveratrol	Protein-based supramolecular NFs	Non-covalent binding; sustained antioxidant release	Oxidative spoilage-related microbes	Sliced apples	~50% shelf-life increase; inhibited enzymatic browning	**[122]**
HP-β-CD	Cinnamaldehyde	Hyaluronic acid active coating	Host–guest IC enabled slow, continuous release	Food-borne microorganisms	Bananas, fresh-cut apples	Reduced weight loss; preserved color and texture	**[123]**

Table S4. Comparative summary of supramolecular and nanofibrous systems for antimicrobial food (meats) preservation.

CD Type	Active Compound(s)	Matrix / Structure	Encapsulation / Loading	Release Behavior	Target Microorganisms	Food Application	Shelf-Life Extension	Key Strengths	Key Limitations	Industrial Readiness	Ref.
β-CD	Citral, trans-cinnamaldehyde	EVOH extrusion film	High; protects volatiles during melt processing	Slow, diffusion-controlled	General spoilage flora	Chilled beef	~4 days	Excellent thermal stability; extrusion-compatible; controlled release	Synthetic polymer; not edible	High	**[124]**
β-CD	OEC	Sodium alginate edible coating	71.6% encapsulation efficiency	Sustained release	*E. coli*, *S. aureus*	Chicken breast	~6 days	Edible, biodegradable; strong antimicrobial + antioxidant	Moisture sensitivity	High	**[125]**
CD (β-CD)	Carvacrol	Chitosan/CD casting film (active pad)	High sorption into food	Rapid + continuous	Broad spoilage microbiota	Chicken fillets	~3–4 days	Strong antimicrobial potency	Severe sensory impact	Low–Moderate	**[128]**

Methyl-β-CD	Thyme oil	PVA–polysaccharide hydrogel	Efficient IC formation	Sustained, Fickian diffusion	*E. coli*, *S. aureus*	Chicken breast	~4 days	Exceptional mechanical strength; controlled release	Hydrogel handling complexity	Moderate–High	**[127]**
— (no CD)	OEO	Dioscorea starch film	Direct loading	Diffusion-driven	*B. subtilis*, *E. coli*, *S. aureus*	Chicken	~5–6 days	Fully biodegradable; strong antibacterial	Limited Gram-negative efficacy	Moderate	**[126]**
— (no CD)	Perilla essential oil	Electrospun PLA NFs	Direct oil loading	Slow migration	General spoilage bacteria	Chicken	~12 days	Excellent barrier + antimicrobial synergy	No volatility control	High	**[129]**
γ-CD	Carvacrol	Electrospun gelatin/pullulan NFs	High retention (≈68% after 2 months)	Controlled release	Gram-negative dominant	Meat, fish oil models	Indirect (oxidation delay)	Superior aroma retention; biodegradable NFs	Electrospinning scale-up	Moderate	**[132]**

β-CD	Corn poppy extract (indicator)	Gelatin/chitosan nanofiber film	Stable IC dispersion	Stimuli-responsive (pH/volatile)	— (indicator)	Shrimp (freshness sensing)	—	Intelligent colorimetric response	Not antimicrobial	Moderate–High	**[133]**
β-CD	Cinnamaldehyde	PET nonwoven antibacterial pad	Stable IC immobilization	Sustained vapor release	*E. coli*, *S. aureus*	Cold fresh pork	~80% increase	Excellent vapor-phase control; minimal sensory impact	Not edible	High	**[131]**
β-CD	— (fat stabilization)	Konjac emulsion gel	Structural role	No release	—	Sausage (fat substitute)	Quality retention	Improves texture & emulsion stability	No antimicrobial function	High	**[134]**
β-CD	Curcumin (photosensitizer)	κ-Carrageenan film	Stable IC dispersion	Light-triggered ROS	Mixed spoilage bacteria	Chilled pork	~5–6 days	Photodynamic, residue-free antimicrobial	Requires light activation	Moderate	**[130]**
